# Sustainable Polymeric Membranes: Green Chemistry and Circular Economy Approaches

**DOI:** 10.1021/acsestengg.5c00282

**Published:** 2025-07-04

**Authors:** Ching Yoong Loh, Andrew D. Burrows, Ming Xie

**Affiliations:** † Department of Chemical Engineering, 1555 University of Bath, Bath BA2 7AY, United Kingdom; ‡ Department of Chemistry, University of Bath, Bath BA2 7AY, United Kingdom

**Keywords:** mechanosynthesis, biopolymer, green solvents, upcycling and downcycling, covalent adaptable network membranes

## Abstract

Water scarcity remains a critical global challenge, necessitating the advancement of sustainable water treatment technologies. Polymeric membranes have emerged as an indispensable solution for desalination and wastewater treatment due to their high efficiency and low energy consumption. However, conventional membrane fabrication relies on petroleum-derived polymers and toxic solvents, generating significant environmental concerns. This review sheds light on the state-of-the-art approaches to sustainable membrane development, focusing on green chemistry principles and circular economy strategies. Mechanosynthesis offers a solvent-free alternative for synthesizing advanced membrane materials, including metal–organic frameworks, covalent organic frameworks, and polymers of intrinsic microporosity. Additionally, the adoption of biobased green solvents, such as Cyrene and γ-valerolactone, provides viable substitutes for hazardous dipolar aprotic solvents traditionally used in membrane fabrication. The incorporation of biopolymers, including cellulose derivatives and polyhydroxyalkanoates, further enhances the sustainability of polymeric membranes. To mitigate membrane waste, circular economy strategies, including downcycling, upcycling, and repreparation via covalent adaptable networks, offer promising pathways for extending membrane lifecycles and minimizing environmental impact.

## Graphical abstract

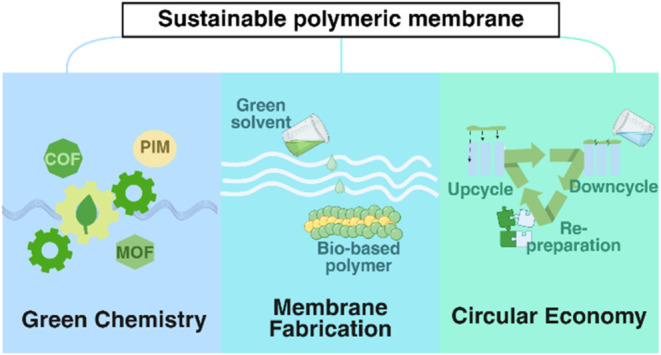

## 1 Introduction

Water scarcity is a pressing global challenge, with approximately 10% of the global population (around 720 million people) living in regions experiencing high or critical water stress levels as of 2021.^1^ The agricultural sector accounts for the majority of global water withdrawals (72%), followed by municipal use for households and services (16%) and industrial activities (12%).^2^ In this context, membrane technology has emerged as a critical solution for mitigating water scarcity by enabling the conversion of saline and contaminated water sources into potable water, aligning with the United Nations’ (UN) Sustainable Development Goal 6 (SDG 6, Clean Water and Sanitation) to ensure availability and sustainable management of water and sanitation for all by 2030. Technologies such as reverse osmosis (RO), nanofiltration (NF), ultrafiltration (UF), and microfiltration (MF) are now integral to desalination and water treatment processes. With nearly 16,000 operational desalination plants worldwide producing approximately 95 million m^3^/day of desalinated water for human use, membrane technology has become indispensable in addressing global water shortages,^3^ and advancing the ambitious targets of SDG 6.

Polymeric membranes, in particular, are widely regarded as a green technology due to their low energy consumption and operational simplicity.^4^ However, the sustainability of membrane fabrication and disposal processes remains a significant concern. Conventional polymeric membranes are predominantly manufactured from petroleum-based polymers such as poly­(vinylidene fluoride) (PVDF), polysulfone (PSf), and poly­(ether sulfone) (PES), which pose considerable ecological challenges.^5^ Furthermore, the fabrication process relies heavily on toxic, dipolar aprotic and petroleum-based solvents like *N*,*N*-dimethylformamide (DMF) and *N*-methyl-2-pyrrolidone (NMP) for phase inversion. This results in the annual generation of over 50 billion liters of wastewater contaminated with these solvents.^6^ The typical lifespan of RO and NF membranes ranges from 3 to 7 years, after which they are often disposed of via landfills or incineration. This practice contributes to significant environmental degradation, with an estimated 14,000 tonnes of RO membranes discarded annually, releasing substantial amounts of CO_2_ into the atmosphere.^7^ These issues highlight the urgent need to re-evaluate conventional membrane fabrication and disposal practices to align with the principles of sustainability and environmental duties.

To address these challenges, an innovative framework integrating green chemistry and circular economy principles has been proposed in this study (Figure 1). Advances in mechanosynthesis (a solvent-free synthesis using mechanical energy), the use of biobased green solvents (e.g., dihydrolevoglucosenone (Cyrene), γ-valerolactone), and renewable biopolymers (e.g., cellulose derivatives, chitosan, biopolyesters) offer a promising pathway for developing high-performance membranes with reduced environmental impact. Concurrently, circular economy strategies such as upcycling, downcycling, and repreparation of end-of-life (EoL) membranes, provide opportunities to extend membrane lifecycles and minimize waste. The growing body of research on sustainable membrane manufacturing for liquid separation underscores the importance of integrating sustainability into membrane fabrication processes (Figure 2). These approaches align with SDG 6, supporting global efforts toward sustainable water management.

**Figure 1:**
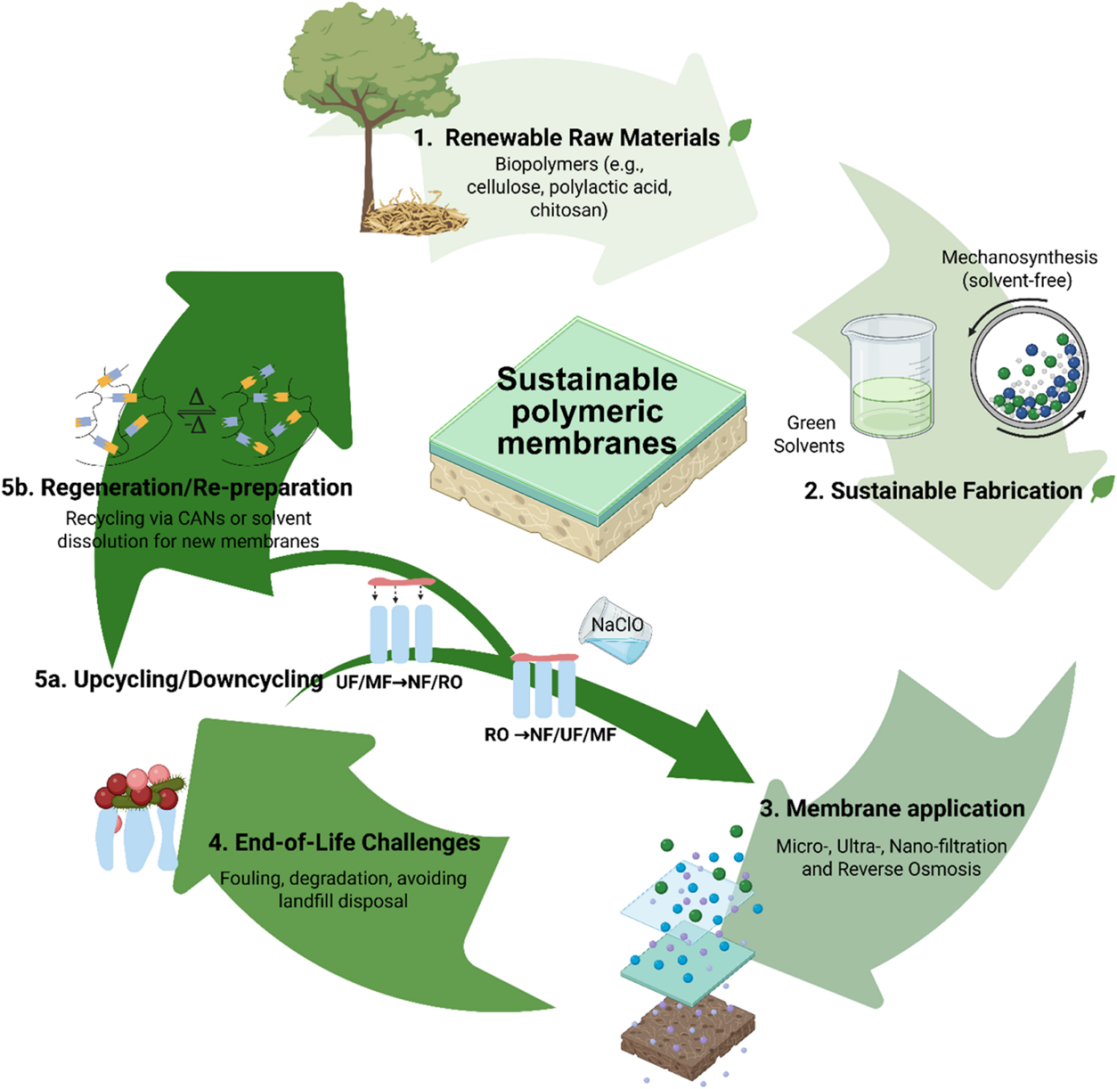
Schematic diagram of the sustainable, closed-loop polymeric membrane fabrication coupling mechanosynthesis, green solvents, biopolymer, and circular economy strategies such as upcycling, downcycling and repreparation of polymeric membranes.

**Figure 2:**
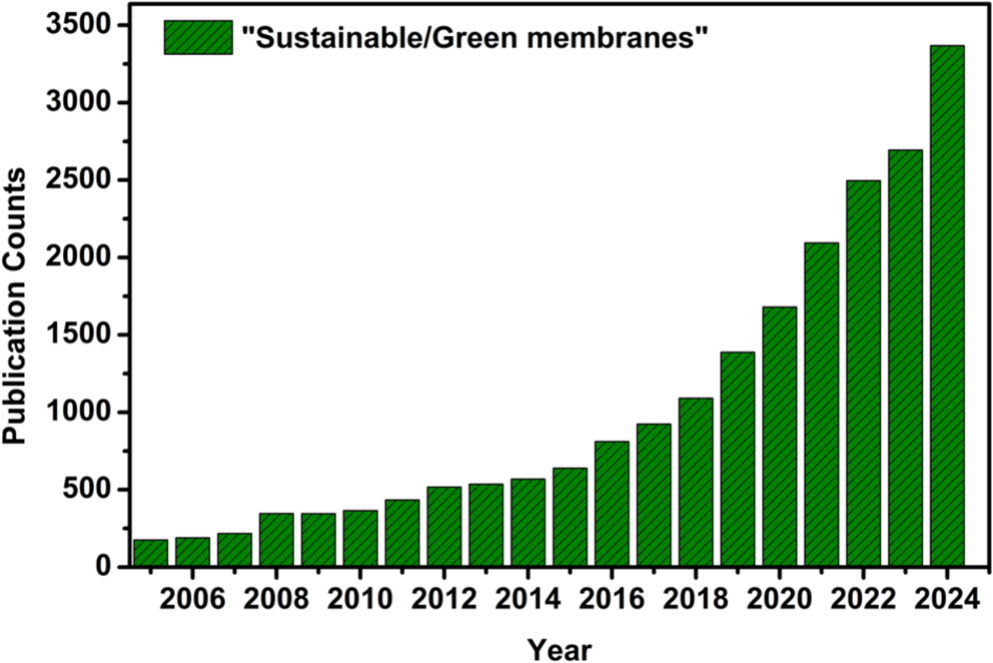
Publication trends on sustainable/green membranes for liquid separation from 2005–2024 (sourced from Web Of Science with the prompt: (“sustainable” OR “green”) AND “membrane” AND “water separation”).

In this review, the state-of-the-art in sustainable membrane fabrication for liquid separation will be critically examined, with a focus on green chemistry and circular economy principles. First, the integration of sustainability into polymeric membrane fabrication is discussed, including the application of mechanosynthesis, green solvents, and biopolymers. Next, the circular economy framework for EoL membranes is explored in detail, emphasizing upcycling, downcycling, and repreparation strategies. Finally, the challenges and future prospects of these technologies are analyzed to provide insights into their scalability and potential impact. By bringing together recent advancements and identifying research gaps, this work seeks to guide the transition toward environmentally responsible membrane technologies that address both water scarcity and ecological sustainability.

## 2 Sustainable Polymeric Membrane Fabrication: Mechanosynthesis, Green Solvent, and Biopolymer

Organic materials have emerged as a dominant class of membrane building blocks for addressing water treatment challenges. Among these, petroleum-based polymers have historically dominated the membrane market due to their cost-effectiveness, flexibility, and robust thermal and chemical stability.^8^ However, the processes associated with crude oil extraction, transportation, and refining pose significant risks of aquatic and atmospheric pollution, contributing to substantial environmental impacts. To address these challenges, it is crucial to advance membrane manufacturing processes by adopting sustainable practices and minimizing environmental footprints. In the current section, we will include the motivation of employing sustainable membrane technologies, the recent trend of mechanosynthesis of membrane materials, the involvement of green solvents in membrane fabrication, biopolymer-based membranes with their economic viability for scaling up, and the current policy and regulatory framework that affects the sustainable membrane technologies.

### 2.1 Motivations for Sustainable Polymeric Membranes

The shift toward sustainable polymeric membrane technologies is driven by pressing global challenges and the need for environmentally responsible solutions in water treatment. First, water scarcity affects over 2 billion people worldwide,^9^ necessitating efficient and sustainable membrane-based processes to provide clean water and meet SDG 6. Additionally, conventional membrane fabrication, reliant on toxic and petroleum-based solvents like NMP and DMF, contributes to environmental pollution, with solvent emissions accounting for significant volatile organic compound (VOC) releases and endangering terrestrial and aquatic life. These traditional membrane fabrication processes also depend on nonrenewable petroleum-based polymers, amplifying resource depletion and greenhouse gas emissions.

In terms of policies, regulatory frameworks, such as the European Union’s Registration, Evaluation, Authorisation and Restriction of Chemicals (REACH) and the U.S. Toxic Substances Control Act (TSCA), are phasing out hazardous chemicals, creating an urgent need for green alternatives like biobased solvents and renewable biopolymers. Economically, sustainable approaches offer long-term benefits, including reduced waste disposal costs and energy savings. Furthermore, the growing demand for circular economy practices, driven by consumer and industry preferences for sustainable products, underscores the importance of recyclable membranes using strategies like upcycling, downcycling and membrane repreparation. By addressing these challenges, sustainable membrane technologies not only mitigate environmental harm but also enhance water security and support economic resilience.

### 2.2 Mechanosynthesis of Organic Membrane Building Blocks

Mechanosynthesis is a synthesis technique that utilizes physiochemical transformations induced by mechanical energy, such as shear, impact, extension, and compression.^10^ Unlike traditional solvothermal methods, mechanosynthesis operates under solvent-free or minimal-solvent conditions, significantly reducing the environmental footprint of chemical processes and aligning with the Twelve Principles of Green Chemistry.^11^ The technique promotes molecular collisions and bond formation through mechanical forces, enabling efficient reactions with reduced energy input and shorter reaction times compared to conventional methods. Key considerations in mechanosynthesis include optimizing reaction efficiency, ensuring reproducibility of material properties (e.g., porosity, surface area), and addressing scalability challenges for industrial applications.

Over the past decade, mechanosynthesis has garnered significant attention from organic chemists and is widely regarded as a green chemistry approach, primarily due to its solvent-free reaction conditions or minimal use of liquid reaction media.^12^ Although mechanochemistry aligns well with the principles of green chemistry, its application in the synthesis of conventional membrane polymers remains limited. This is primarily due to the mechanical forces involved, such as grinding and shearing, which induce external stress on polymer chains, often resulting in chain scission and stretching.^13–15^ These effects reduce molecular weight, viscosity, and mechanical strength,^16^ properties critical for effective membrane fabrication. As high molecular weight is vital for achieving robust membrane-forming capabilities and mechanical performance, these limitations hinder the broader application of mechanochemistry in conventional polymer synthesis.

In contrast, novel organic porous materials such as metal–organic frameworks (MOFs), covalent organic frameworks (COFs), and polymer of intrinsic microporosity (PIMs) offer promising alternatives for membrane fabrication due to their high specific surface areas, chemical and thermal stability, and tunable functional groups. However, the conventional synthesis of these materials often involves significant quantities of toxic and hazardous solvents and extended reaction times. For instance, the Innovative Medicines Initiative (IMI)–CHEM 21 has classified toluene and dimethylacetamide (DMAc) as hazardous and problematic, respectively,^17^ both of which are commonly used as reaction media in traditional PIM-1 synthesis.^18–20^ Numerous studies have reported successful mechanosynthesis of novel organic porous materials in the past years (Table 1), making mechanochemistry a viable option to substitute traditional synthesis. Therefore, this subsection focuses on recent advancements in the mechanosynthesis of membrane building blocks as a more sustainable approach (Figure 3).

**Figure 3:**
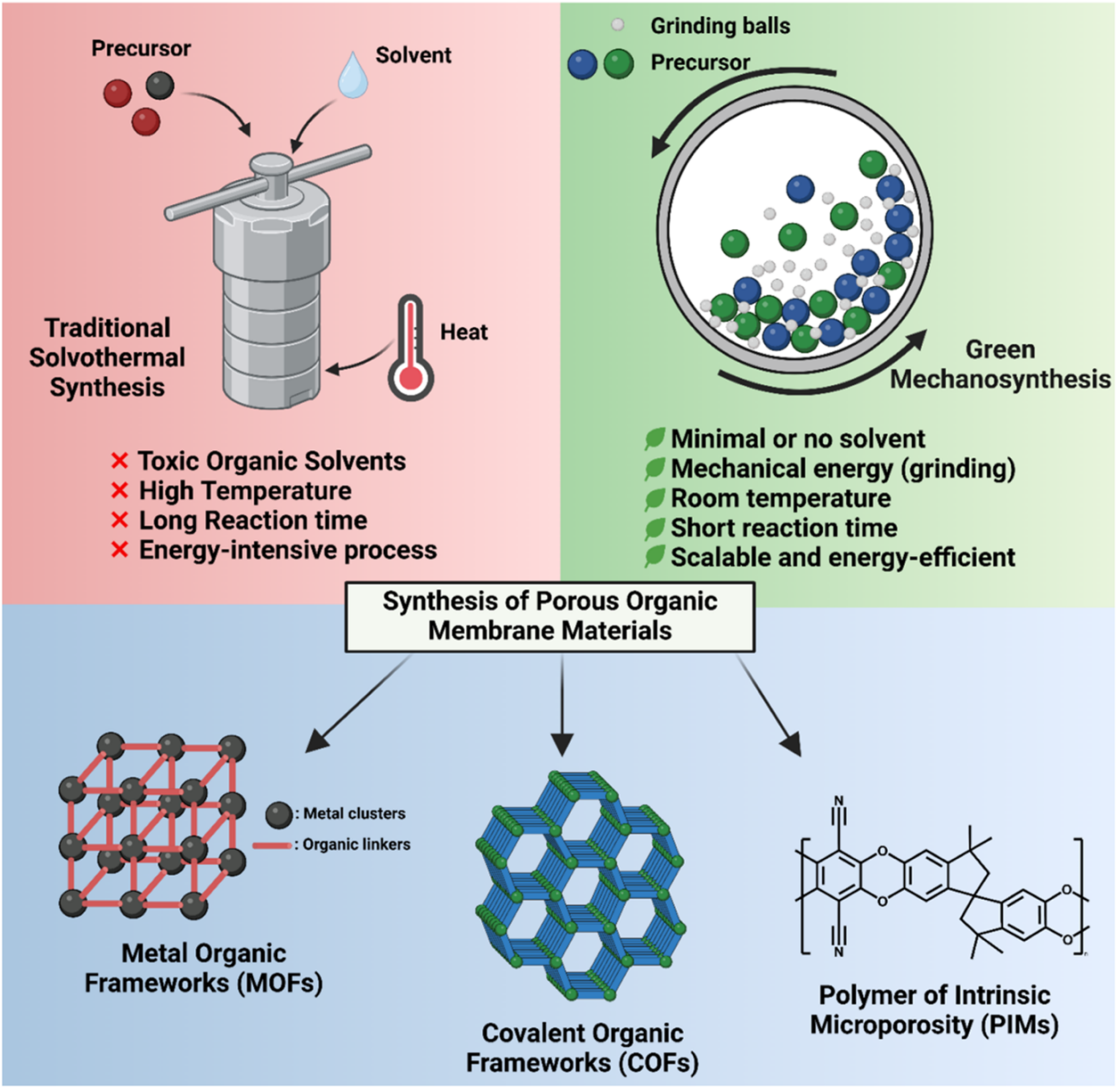
Schematic diagram for the comparison of traditional solvothermal synthesis and mechanosynthesis for producing organic membrane materials such as MOFs, COFs, and PIMs.

**Table 1:** Recent Studies on Mechanosynthesis of Organic Membrane Building Blocks with Their Environmental Implications and Performance

	name	mechanosynthesis method	reaction time (min)	yield	additive/solvent used	*S*_BET_^a^ (m^2^/g)	refs
metal–organic framework	CuBTC	planetary mill	60	50–60%		1638	^25^
	MOF-303	planetary mill	180	50–60%	H_2_O	1180	^25^
	MOF-801	mixer mill	90	87 mg	H_2_O	540	^24^
	MOF-804	mixer mill	90	108 mg	H_2_O	755	^24^
	UiO-66	mixer mill	120	95 mg	H_2_O	1145	^24^
	UiO-66-NH_2_	mixer mill	90	111 mg	H_2_O	815	^24^
	UiO-66-NH_2_	planetary mill	90	11.3 g	H_2_O	885	^24^
	UiO-66-NH_2_	twin screw extrusion	1.4 kg/h		methanol	610	^24^
	ZIF-8	planetary mill	70	50–60%		1609	^25^
	ZIF-9	hand-grinding		∼10 mg	ethanol		^22^
	ZIF-UC-6	mixer mill	30		DMF	504	^23^
covalent organic framework	COF-1	mixer mill	45	76 mg	mesitylene + dioxane/THF	680	^41^
	COF-102	mixer mill	45	50 mg	mesitylene + dioxane/THF	2600	^41^
	DMTP-TPB	mixer mill	60	0.9 g, 83%	acetic acid + acetonitrile	1554	^42^
	Tp+Azo	hand-grinding	15		*p*-toluenesulfonic acid	1707	^31^
	Tp+BD-(SO_3_H)_2_	hand-grinding	15	72%		37	^29^
	Tp+Pa-1+MCNT	hand-grinding	25		*p*-toluenesulfonic acid + H_2_O	218	^30^
	Tp+Pa-1+nanocellulose	ball mill	20		*p*-toluenesulfonic acid + H_2_O	247	^43^
polymer of intrinsic microporosity	PIM-1	mixer mill	15	0.9 g, 98%		520	^38^
	PIM-1	planetary mill	20			545	^39^
	PIM-1	planetary mill	60			856	^39^
	PIM-4	mixer mill	15			179	^38^

a *S*_BET_: Brunauer–Emmett–Teller (BET) surface area.

#### 2.2.1 Metal–organic Frameworks (MOFs)

MOFs are a class of porous materials composed of individual metal centers or clusters linked by bridging organic ligands, offering unique advantages such as a uniform porous structure, large specific surface area, and abundant functional groups,^21^ making MOFs particularly attractive for membrane fabrication. The feasibility of MOF mechanosynthesis has been successfully demonstrated by various researchers.^22–25^

Mechanochemistry is a great synthesis tool for MOFs, but it can also alter the dimensions of the material. Saini and colleagues synthesized a two-dimensional (2D) cobalt-based zeolitic imidazolate framework, ZIF-9-III, using a mechanochemical process and subsequently incorporated it into a composite membrane (ZIF-9@PVDF) via a nonsolvent-induced phase inversion (NIPS) process.^22^ The mechanochemical synthesis involved manual grinding with a minimal amount of ethanol, whereas the conventional solvothermal method required toxic organic solvents, such as DMF, and high energy input (130 °C for 48 h). Interestingly, the two synthesis methods yielded distinct structural outcomes. The conventional solvothermal method produced ZIF-9-I with a three-dimensional (3D) sodalite structure characterized by large open pores, while the mechanochemical approach exfoliated ZIF-9-III into 2D nanosheets. This structural difference significantly influenced the materials’ performance in oil/water separation. Mechanochemically synthesized ZIF-9-III exhibited superhydrophobic properties (water contact angle: 144°) and achieved superior separation efficiency (removal efficiency: 99.8%) compared to conventionally synthesized ZIF-9-I (water contact angle: ∼92°).

The scalability of a technology is equally important to its efficiency and performance. Karadeniz’s group successfully developed a scalable water-assisted mechanosynthesis process for zirconium-based MOFs (UiO-66, UiO-66-NH_2_, MOF-801, and MOF-804), achieving good yields after reaction times of 30–90 min.^24^ The synthesized MOFs demonstrated comparable BET surface areas to those produced via solvothermal methods, maintaining the crucial characteristics of the material while eliminating the use of toxic organic solvents and reducing energy use for long reaction time. Importantly, the water-assisted mechanosynthesis achieved scalability, with 10 g-scale production using a laboratory planetary mill and over 100 g-scale production of UiO-66-NH_2_ via twin-screw extrusion technology, achieving a throughput of 1.4 kg/h. Therefore, this study revealed a potential scalable approach for mechanosynthesizing MOFs using twin-screw extrusion.

#### 2.2.2 Covalent Organic Frameworks (COFs)

COFs are an emerging class of crystalline, porous materials composed entirely of light elements (H, C, O, N, and B) bonded covalently. Their low density, inherent porosity, large surface area, and tunable pore sizes make them appealing for membrane applications.^26–28^ Recent advancements in mechanosynthesis provide a promising pathway to greener COF fabrication, circumventing the need for hazardous solvents and potentially reducing energy input.

Lin et al. reported a dual-functional COF with high adsorption capacity and fluorescence sensing ability, synthesized using a mortar and pestle with 2,4,6-triformylphloroglucinol (Tp) and 2,2′-benzidinedisulfonic acid (BD-(SO_3_H)_2_) as precursors.^29^ The resulting TpBD-(SO_3_H)_2_ COF provided abundant negatively charged adsorption sites, achieving a maximum norfloxacin adsorption capacity of 1709 mg/g. This facile and green mechanochemical synthesis highlighted its potential for environmental applications.

Aside from synthesizing pure COFs, Liu et al. fabricated a magnetic COF composite using mechanochemical reaction to remove microcystins in lake water.^30^ The facile mechanosynthesis involves a one-step hand-grinding of Tp, *p*-phenylenediamine (Pa-1) and carbon nanotubes encapsulated magnetic nanoparticles (MCNTs), where the magnetic COF composite exerts a high recovery of microcystins (>85%). Interestingly, this research opens up opportunities to prepare magnetic COFs with excellent properties via green mechanochemistry.

The mechanosynthesis-enabled COFs are not limited to water treatment membrane processes. Wu and colleagues fabricated a freestanding COF membrane by reacting Tp and 4,4′-azodianiline (Azo) via Schiff base reactions using a hand-grinding method.^31^ The membrane was subsequently modified with sulfonated poly­(ether ether ketone) (s-PEEK) for application in flow batteries as a proton exchange membrane, achieving high proton conductivity (10.88 mS/cm). This innovative approach demonstrated the potential of mechanochemistry in fabricating greener membranes with excellent performance.

#### 2.2.3 Polymers of Intrinsic Microporosity (PIMs)

PIMs are a class of highly porous organic materials characterized by rigid, contorted polymer backbones that create interconnected micropores. These unique structural attributes, including high surface area, excellent permeability, and solution processability, make PIMs highly promising for applications in gas separation, water treatment, and organic solvent nanofiltration (OSN).^32–36^ Among the various PIMs, PIM-1 stands out as one of the most widely studied and utilized archetypes. Its synthesis typically involves the step-growth polymerization of 5,5′,6,6′-tetrahydroxy-3,3,3′,3′-tetramethyl-1,1′-spirobisindane (TTSBI) with tetrafluoroterephthalonitrile (TFTPN) in a solvent system comprising DMAc and toluene at 160 °C for approximately 60 min.^37^ However, this approach heavily relies on toxic solvents, such as DMAc, which pose significant environmental and health hazards. Therefore, developing greener synthesis routes for PIM-1 that align with the principles of green chemistry and sustainability is imperative.

Dai’s group demonstrated an innovative synthesis of PIM-1 using mechanochemistry.^38^ By employing ball-grinding, the reaction time was significantly reduced to 15 min compared to the 24–72 h required for conventional PIM-1 synthesis. The external mechanical force generated during ball milling enhances molecular collisions, facilitates rapid chain growth, and minimizes premature chain termination. Consequently, the mechanochemically synthesized PIM-1 exhibits a higher weight-average molecular weight (*M*_w_: 485,000 g/mol) and a low polydispersity index (*M*_w_/*M_n_*: 1.4). Moreover, the resulting PIM-1 is readily soluble in organic solvents such as tetrahydrofuran (THF) and chloroform, improving its processability and broadening its potential applications.

In addition to synthesis efficiency and material properties, assessing the environmental impact of mechanochemical approaches is crucial to ensure sustainability. Loh et al. synthesized PIM-1 using a planetary ball mill, achieving a reaction time of under 60 min.^39^ The BET surface area (*S*_BET_) of the mechanochemically synthesized PIM-1 exceeded 800 m^2^/g, falling within the typical range for PIM-1 (*S*_BET_: 500–1000 m^2^/g).^40^ Furthermore, Xie’s group conducted a Life Cycle Assessment (LCA) comparing mechanochemical synthesis with the conventional solvent-based method. The LCA revealed that the environmental impact of conventional synthesis is 1.5 times greater than that of mechanochemical synthesis for PIM-1 production.^39^

#### 2.2.4 Economic Viability of Mechanochemistry

The economic viability of a technology is a critical factor influencing its potential for commercialization. Techno-economic analysis (TEA) is frequently employed as a robust tool to assess the commercial feasibility of emerging technologies. In a recent study, Wenger et al. conducted a TEA on the green mechanochemical synthesis of UiO-66-NH_2_.^44^ Using a batch mechanosynthesis approach via ball milling, they achieved a production rate of 3.01 g/h with a 43% yield. To improve the accuracy of their cost estimation, the authors incorporated labor costs into the analysis, resulting in a levelised production cost of approximately USD 6,498 per kilogram of UiO-66-NH_2_. This figure is notably lower than the current market price offered by commercial MOF producers, which exceeds USD 10,000 per kilogram.^44^ These findings highlight mechanosynthesis not only as an environmentally sustainable method but also as an economically competitive alternative for MOF production.

Energy consumption is also a fundamental factor in evaluating the economic viability of a technology, as it directly influences operational costs. Loh et al. compared the energy requirements for producing PIM-1 using mechanosynthesis and conventional wet chemical methods, reporting energy consumptions of 24.6 and 36.9 kWh/g, respectively.^39^ While this comparison is based on laboratory-scale synthesis, it clearly shows the significant difference in energy usage between the two methods. Specifically, the mechanochemical route consumes approximately 1.5 times less energy than the conventional method, indicating great potential for energy savings during scale-up of PIM-1 production and hence better economic value.

Despite its potential as a green and solvent-free synthesis route, mechanochemistry faces a significant challenge in terms of capital investment. A notable cost disparity exists between conventional hydrothermal synthesis and mechanosynthesis, particularly at the laboratory scale. For example, a planetary ball mill (e.g., RETSCH PM 100) typically costs around £10,000, whereas a hydrothermal setup, comprising a 100–500 mL Teflon-lined autoclave (£100–£1000) and a laboratory oven (approximately £2,000), requires substantially less investment. This 3-fold difference in cost makes mechanosynthesis less accessible, especially for small- and medium-sized enterprises. Therefore, to enhance its practical viability, further attention must be given to optimizing process parameters to improve both energy efficiency and product yield.

### 2.3 Green Solvents in Membrane Fabrication Processes

Having explored mechanosynthesis, we now turn to green solvents as a complementary strategy. As mentioned, one of the governing class of material used in membrane fabrication is represented by polymers, for example, polyacrylonitrile (PAN), PSf, PES, polyimide (PI), polyamides (PAs), cellulose and fluoropolymers.^45^ Commercial polymeric membranes are predominantly produced using the phase inversion method—a controlled process that transitions a polymer from a viscous liquid to a solid state.^46–48^ This transformation is driven by polymer precipitation, induced by various mechanisms such as nonsolvent immersion, vapor phase exposure, solvent evaporation, or thermal changes. Among the key factors influencing membrane characteristics, the choice of solvent for polymer dissolution plays a critical role, directly affecting membrane properties, including morphology, porosity, mechanical strength, and pore size. These structural attributes, in turn, dictate performance metrics such as permeability and selectivity.^46,49^

Traditionally, membrane fabrication has relied on toxic organic solvents and petroleum-based compounds, such as NMP, DMAc, DMF, and THF.^50,51^ Notably, this manufacturing process generates over 50 billion liters of wastewater annually, contaminated with hazardous solvents.^6^ To address these environmental and health concerns, recent years have seen the emergence of alternative solvents with lower toxicity and reduced environmental impact (Table 2). A summary of the solvent characteristics is illustrated in Figure 4. This section focuses on these green solvents, which hold promise for sustainable membrane fabrication processes.

**Figure 4:**
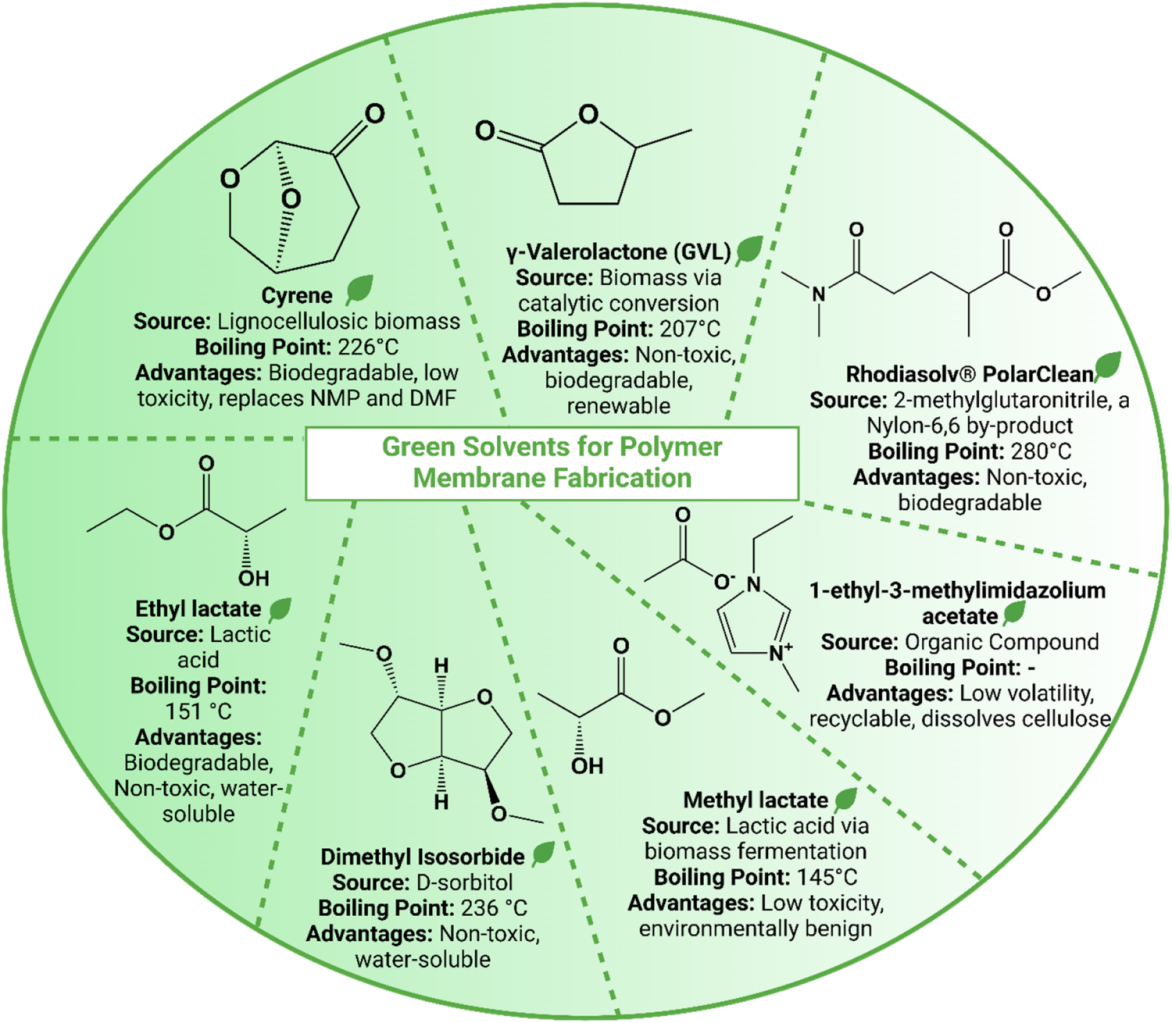
Summary of green solvents for polymer membrane fabrication, highlighting sustainable alternatives to conventional solvents. The diagram includes solvents such as Cyrene, GVL, PolarClean, [EMIM]­OAc, methyl lactate, DMI, and ethyl lactate, detailing their natural or renewable sources, boiling points, and eco-friendly advantages, for example, biodegradability, low toxicity, and water solubility.

**Table 2:** Comparison between the Recent Studies on Sustainable Membrane Fabrication via Green Solvents and Petroleum-based/Toxic Solvents

	solvents	polymer	cosolvent	membrane fabrication method	application	flux/permeance^a^	rejection^a^	refs
green solvents	cyrene (biobased)	matrimid	THF	NIPS	gas separation			^81^
		PES		spray coating + NIPS	UF, support layer	68.9		^55^
		PLA		NIPS	UF	24.0		^72^
		PSf		NIPS	UF	29.1	BSA: 96.7%	^54^
		PVDF		NIPS	UF			^82^
	dimethyl isosorbide (biobased)	PES		VIPS + NIPS	UF	2500		^50^
		PLA		NIPS	UF	389		^72^
		PVDF		VIPS + NIPS	UF	2100		^50^
	ethyl lactate (biobased)	CA	ethanol	electrospinning	photosensitizer			^83^
		CA + GO		NIPS	UF	6.0	methyl orange: 38.9%	^84^
		PLA		NIPS	UF	93.0		^72^
	[EMIM]OAc (synthetic)	cellulose	acetone	NIPS	oil/water separation	39.0	emulsion retention: 99.0%	^74^
		cellulose	DMSO + H_2_O	NIPS	gas separation			^85^
		CA	acetone	NIPS	UF	110.0–330.0	BSA: 91.0%	^75^
							γ-globulin: 96.0–98.0%	
	methyl lactate (biobased)	CA	2-MeTHF	NIPS	NF	2.4–12.8	rose Bengal: 93.0–99.5%	^70^
		CA		NIPS	NF	0.2	100 kDa PEG: 100%	^86^
		PLA		NIPS	UF, MF	21.9	10 kDa dextran: 99.9%	^87^
	rhodiasolv PolarClean (biobased)	PSf		NIPS	UF, support layer	4000		^67^
		PVDF-HFP		VIPS/NIPS	UF	4000–10,000	methylene blue: 72–89%	^68^
	γ-valerolactone (biobased)	CA		NIPS	NF	1.7	rose Bengal: 96.2	^61^
		CTA		NIPS	NF	15.9	rose Bengal: 94%	^61^
		polyimide		NIPS	NF	2.6	rose Bengal: 97.2%	^61^
		PES		NIPS	NF	1.1	rose Bengal: 96.2%	^61^
		PSf		NIPS	UF, support layer	5,500		^67^
		PSf		NIPS	UF	15.6	BSA: 92.7%	^54^
		PSf		NIPS	NF	1.3	rose Bengal: 98.3	^61^
petroleum-based/toxic solvents	DMAc	PES		NIPS	UF	44.4	powder milk proteins: 98%	^88^
		PSf		NIPS	UF	10.7	BSA: 90.1%	^89^
		PSf		NIPS	UF	15.8	BSA: 96.0%	^90^
		PVDF		NIPS	UF	32.9	protein: 98.0%	^91^
	DMF	PSf		NIPS	UF	3.2	BSA: 95.1%	^89^
		PSf		NIPS	UF	55.8	BSA: 95.2%	^92^
		PVDF		NIPS	UF	17.0	BSA: >80%	^93^
	NMP	CA		NIPS	UF	49.3		^94^
		CA	γ-butyrolactone	NIPS	UF	1,78l	100k dextran: 90%	^95^
		PES		NIPS	UF	39.4		^96^
		PSf		NIPS	UF	46.3	BSA: >96.0%	^97^
		PVDF		NIPS	UF	9.2	river water TOC removal: 73%	^97^

a Data is only referenced for water separation membranes.

#### 2.3.1 Green Solvent Alternatives

##### 2.3.1.1 Cyrene

Cyrene is a chiral, bicyclic cycloalkane (Figure 4), which is a waste-derived and fully biodegradable aprotic dipolar solvent. It is an alternative solvent to the petroleum-based NMP and DMF, developed by Sherwood et al.^52^ The green solvent has a boiling point of 226 °C and high water solubility. Polymer materials such as PES, PVDF and cellulose acetate (CA) are highly dissolvable in Cyrene. Membranes fabricated using Cyrene typically exhibit sponge-like^53,54^ or finger-like^55^ pore structures in the support layer, depending on the polymer and coagulation bath conditions. These morphologies closely resemble those of membranes produced using conventional solvents, indicating that Cyrene is a viable substitute in terms of membrane structure and formation characteristics.

To further assess the viability of Cyrene, a comparison test between conventional solvent and green alternative is necessary. Lau’s group employed Cyrene in the fabrication of PES ultrafiltration membranes.^55^ They demonstrated that PES ultrafiltration membranes fabricated via spray coating using Cyrene possess better performance than those prepared using the traditional knife cast method using NMP as the solvent, reaching a membrane permeance of 68 L/m^2^·h·bar, 7 times higher than the knife cast membrane. In addition to increasing membrane performance, the environmental impact of the membrane fabrication process was also reduced due to the usage of greener solvents.

However, a comprehensive assessment of Cyrene must consider its environmental footprint alongside its performance benefits. Also conducted by Lau’s group, the LCA have indicated that membranes produced using biorenewable solvents such as Cyrene may initially exhibit a higher environmental impact than those made with traditional solvents.^56^ This is primarily due to lower membrane yields and the limited scope of early stage assessments. Nevertheless, the higher permeance of green solvent-based membranes may offset these drawbacks during later stages of the product life cycle, leading to lower operational energy requirements and, ultimately, a reduced overall environmental burden.

##### 2.3.1.2 γ-Valerolactone (GVL)

γ-Valerolactone (GVL) is another polar aprotic solvent (Figure 4). It is a popular nontoxic solvent with high boiling point (207 °C) and has been used in chemical processes and as a flavor additive in perfumes.^57,58^ In a typical synthetic process, biomass, such as lignocellulose, can be directly converted into GVL using a one-pot catalytic system, integrating all hydrolysis, dehydration, and hydrogenation processes. The steps included hydrolysis of cellulose or hemicellulose to sugar, dehydration of sugar to form furfural or levulinic acid and later formation of GVL from the hydrogenation of furfural and levulinic acid.^59^

In membrane fabrication, GVL has demonstrated promising results as an alternative to petroleum-based solvents such as NMP. Wang’s group reported that membranes fabricated using GVL exhibit a sponge-like porous structure, contrasting with the finger-like structures often associated with NMP-based membranes.^60^ This morphological difference is attributed to the disparity in solubility parameters between the solvent and water: GVL (17.5 MPa^1/2^) has a significantly greater mismatch with water (47.8 MPa^1/2^) compared to NMP (23.3 MPa^1/2^). The reduced compatibility between GVL and water lowers the thermodynamic driving force for water in-diffusion during phase inversion, promoting sponge-like pore formation.

By using GVL as the green alternative solvent for NIPS method, Rasool and Vankelecom have managed to fabricate NF membranes with different polymeric materials, including CA, PES, PSf, PI and cellulose triacetate (CTA).^61^ All polymers yield reasonable permeance and high rejection toward a cationic dye, Rhodamine B (RB). The best-performing membrane material when using GVL as solvent is CTA, with a high permeance of 16 L/m^2^·h·bar and excellent RB-rejection of 94%. This study highlights the potential of green solvents for tight NF membranes and demonstrates their applicability in fabricating ultrafiltration membranes or support layers for thin-film composites.

While green solvents such as GVL are generally assumed to provide environmental benefits, LCA studies have challenged this assumption. Lu et al. conducted an LCA comparing the environmental impacts of membranes fabricated using GVL and NMP.^62^ Similar to the case in Cyrene-based membrane, their findings revealed that PSf membranes fabricated with GVL incurred higher environmental burdens in most impact categories compared to those made with NMP. These results highlight that bioderived solvents do not necessarily yield lower environmental footprints, as secondary materials and emissions associated with their production can offset their ecological advantages.

##### 2.3.1.3 Rhodiasolv PolarClean

Methyl-5-(Dimethylamino)-2-Methyl-5-Oxopentanoate (commonly known as PolarClean, Figure 4) is a nonionic, water-soluble, biodegradable, and eco-friendly synthetic organic solvent. PolarClean has been reported to pose no health hazards during PVDF membrane casting, making it a promising alternative to traditional solvents.^63,64^ Commercially available from Solvay Novecare, PolarClean is derived from the valorisation of 2-methylglutaronitrile, a byproduct generated during the synthesis of Nylon-6,6.^65,66^ Fionah et al. successfully demonstrated the use of PolarClean in the fabrication of biochar-incorporated PSf support layer membranes.^67^ Compared to membranes synthesized using NMP, which exhibits a finger-like pore structure, PolarClean-fabricated membranes displayed a sponge-like pore structure, enhancing surface wettability. Additionally, membranes synthesized with PolarClean showed higher leaching compared to their NMP counterparts.

Russo’s group further explored the application of PolarClean in the fabrication of flat-sheet poly­(vinylidene fluoride-hexafluoropropylene) (PVDF-HFP) membranes via vapor-induced phase inversion (VIPS) and NIPS.^68^ The resulting PVDF-HFP membranes demonstrated a high water permeance of up to 3200 L/m^2^·h·bar and an excellent rejection efficiency for methylene blue (MB) dye at 89%. Their study also highlighted the ability to control critical membrane properties (surface roughness, morphology, and pore size) by modifying parameters including the use of pore-forming additives and variations in the coagulation bath composition.

##### 2.3.1.4 Lactate Esters

Methyl lactate (Figure 4), a biodegradable derivative of lactic acid, is a versatile solvent with the ability to dissolve PVDF and CA polymers.^69,70^ By tuning the types of polymer and polymer concentration, sponge-like^70^ and finger-like^70,71^ pore structure of the membrane can be achieved using methyl lactate as the solvent, showing the versatility of the green solvent. Rasool et al. successfully utilized methyl lactate as the primary solvent to fabricate an NF-based CA membrane.^70^ The resulting membrane demonstrated a high rejection efficiency of 92% for rose Bengal dye, with a permeance of 3.5 L/m^2^·h·bar. Additionally, the membrane achieved efficient removal of magnesium sulfate (MgSO_4_), with a rejection rate of 97% and permeance of 1.3 L/m^2^·h·bar. These results underscore the potential of green solvents like methyl lactate to facilitate high-performance membrane fabrication while reducing environmental impact. However, it is important to note that the CA membrane fabricated in this study did not surpass the performance of commercially available membranes, highlighting an area for further optimization.

Ethyl lactate (Figure 4), were also explored for polylactic acid (PLA) membrane fabrication.^72^ Ethyl lactate, synthesized through the esterification of ethanol and lactic acid derived from biomass via fermentation, is recognized as a sustainable solvent. It exhibits desirable properties such as low volatility, low viscosity, and a high boiling point of 154 °C, alongside its environmentally benign nature. These characteristics underscore its potential as a safe and effective alternative for membrane fabrication processes. Interestingly, the membrane morphology of the ethyl lactate-based PLA membrane shows a finger-like macro-void structure, which is similar to employing Cyrene and dimethyl isosorbide (DMI) green solvents.^72^

##### 2.3.1.5 Ionic Liquids

Ionic liquids (ILs) are liquid salts with melting points below 100 °C, typically composed of a bulky, asymmetric organic cation paired with a weakly coordinating organic anion (Figure 4). Although they are synthetic solvents, they possess green properties, such as recyclable and low volatility. ILs have been extensively used as solvents for cellulose, one of the most abundant natural polymers, which presents significant challenges in dissolution. Cellulose membranes are often fabricated via the NIPS method, a process facilitated by the high miscibility of ILs with water.^73^ Chen et al. demonstrated the successful use of 1-ethyl-3-methylimidazolium acetate ([EMIM]­OAc) as a solvent for cellulose-based membranes in oil–water separation.^74^ The incorporation of acetone as a cosolvent significantly enhanced the water permeability of the membrane by 178%, while maintaining a high emulsion retention efficiency of 99.0%.

In addition to dissolving pure cellulose, the ionic liquid [EMIM]­OAc can also dissolve cellulose derivatives such as CA. Kim et al. employed [EMIM]­[OAc] as the primary solvent in the fabrication of CA ultrafiltration (UF) membranes.^75^ The resulting membrane morphology varied significantly, ranging from sponge-like pore structures to macrovoids, depending on the presence of a cosolvent and the concentration of the polymer. The CA membranes fabricated using [EMIM]­[OAc] exhibited promising separation performance, achieving a permeance of 110 L m^–2^ h^–1^ bar^–1^ and a protein rejection rate of up to 98%. This performance is comparable to that of CA membranes prepared using conventional NMP, which typically demonstrate a permeance of 100 L m^–2^ h^–1^ bar^–1^ and similarly high protein rejection. These findings suggest that [EMIM]­[OAc] is a viable green solvent alternative for CA membrane fabrication, offering competitive separation efficiency with potentially reduced environmental impact.

Despite their advantages, the use of ILs is not without drawbacks. Their high production costs, energy-intensive synthesis processes, and potential environmental impact undermine their reputation as green chemicals. Studies have reported the toxicity of certain ILs and their slow biodegradability, raising concerns about their suitability as sustainable alternatives to conventional solvents.^76–80^ Therefore, the adoption of ILs as green solvents requires careful evaluation of their environmental and economic implications.

##### 2.3.1.6 Other

Dimethyl isosorbide (DMI, Figure 4) is a nonhazardous, water-soluble solvent derived from D-sorbitol, a renewable sugar-based starting material. It has emerged as a promising green alternative to many dipolar aprotic solvents. DMI-based membranes possess similar morphological structure as of Cyrene-based membranes when using PLA as the polymeric material. The DMI-based PLA membrane presents a macrovoid with finger-like pore structure in the support layer, similar to the Cyrene- and ethyl lactate-based PLA membrane.^72^

Employing DMI on membrane fabrication also provide good filtration performances. Gomez d’Ayala et al. demonstrated the sustainable fabrication of PLA membranes using DMI, which exhibited excellent properties.^72^ The membranes had pore sizes ranging from 0.02 to 0.09 μm and achieved a pure water permeance of 314 L/m^2^·h·bar, highlighting their high performance.

#### 2.3.2 Compatibility of Green Solvents

While these green solvents show promise, their practical adoption hinges on compatibility with membrane polymers. Hansen solubility parameters (HSP) theory is a powerful tool for predicting polymer solubility in solvents to achieve uniform solutions.^57,98,99^ HSP theory is based on three key physiochemical components of molecular interactions: dispersive interactions (van der Waals’ force, δ_d_), polar interactions (δ_p_) and hydrogen bonding (δ_h_). Table 3 shows the HSP values for various solvents. Using these parameters, the Hansen solubility sphere can be modeled to determine the compatibility of a solvent for dissolving a specific polymer. This compatibility is assessed by calculating the interaction distance (*R*_a_) between the polymer and the solvent, as described by eq 1

1 $$R_{a}=\sqrt{4\times \left({\left(\delta_{d,P}-\delta_{d,S}\right)}^{2}+{\left(\delta_{p,P}-\delta_{p,S}\right)}^{2}+{\left(\delta_{h,P}-\delta_{h,S}\right)}^{2}\right)}$$

where P and S are the polymer and solvent, respectively.

**Table 3:** HSP Values for Various Solvents

	Hansen solubility parameters			
solvents	δ_d_ (MPa)	δ_p_ (MPa)	δ_h_ (MPa)	refs
water	15.6	16.0	42.3	^51^
DMF	17.4	13.7	11.3	^51^
NMP	18.0	12.3	7.2	^51^
rhodiasolv PolarClean	15.8	10.7	9.2	^51^
cyrene	18.8	10.6	6.9	^51^
dimethyl isosorbide	17.6	7.1	7.5	^51^
methyl lactate	15.5	7.2	7.6	^57^
ethyl lactate	16.0	7.6	12.5	^100^
γ-valerolactone	17.1	11.9	6.2	^51^

The Hansen solubility sphere is constructed by defining a radius (*R*_0_) that corresponds to the maximum solubility distance from the polymer center in a three-dimensional (3D) space (Figure 5). A solvent with a lower *R*_a_ value indicates better compatibility with the polymer and a higher likelihood of forming a homogeneous and stable doped solution. Conversely, if *R*_a_ exceeds *R*_0_, the solvent is considered a bad or undesirable solvent to be used for dissolving the polymer.

**Figure 5:**
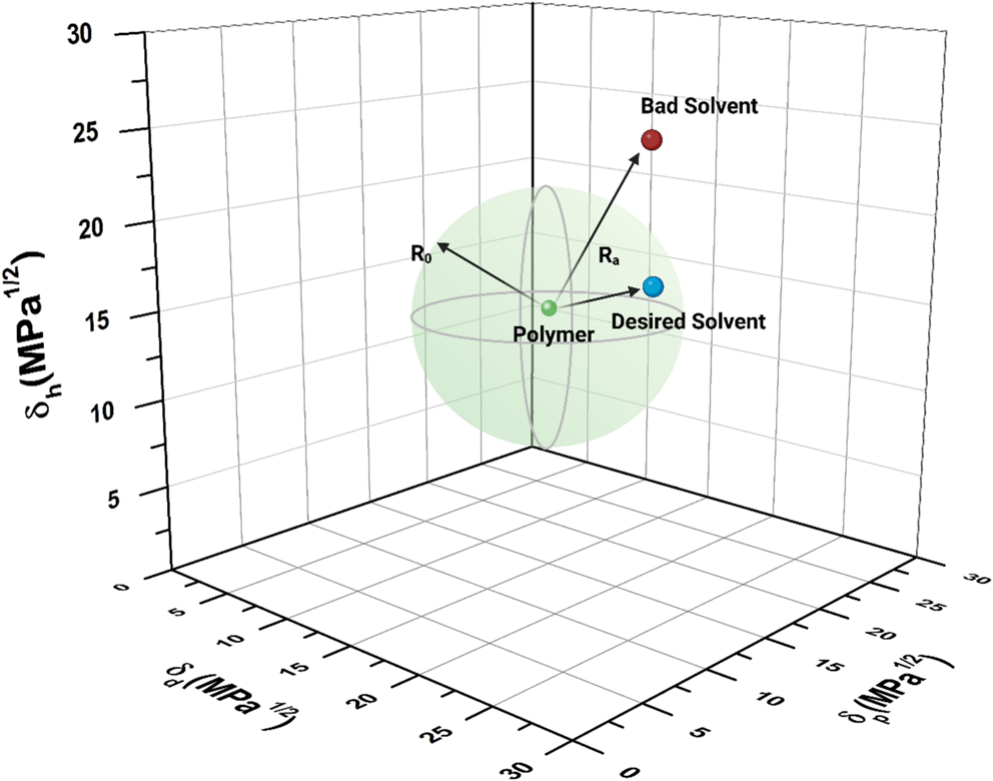
Hansen solubility sphere (green sphere) and *R*_a_ using a three-dimensional graph. The green dot at the center denotes the polymer’s solubility parameters, with the sphere’s radius (*R*_0_) defining the solubility boundary. The blue dot (Desired Solvent) lies within the sphere (*R*_a_ < *R*_0_), indicating good compatibility, while the red dot (Bad Solvent) lies outside (*R*_a_ > *R*_0_), indicating poor compatibility. *R*_a_ represents the distance between the polymer and solvent in solubility parameter space.

Furthermore, the miscibility of the polymer in a solvent can be determined by calculating the relative energy difference (RED), defined as

2 $$\mathrm{RED}=\frac{R_{a}}{R_{0}}$$

The RED value provides insights into solvent performance. If RED < 1, the solvent is considered good, exhibiting high miscibility with the polymer. When RED > 1, the solvent is unsuitable, leading to a nonhomogeneous polymer mixture. In rare cases, when RED = 1, the solvent is at a boundary condition and may only marginally dissolve the polymer.

#### 2.3.3 Economic Challenges in Adopting Green Solvents for Membrane Fabrication

The shift toward green solvents in polymeric membrane fabrication is driven by environmental and health considerations. However, economic viability remains a major barrier to widespread adoption. While green solvents are often biodegradable, nontoxic, and derived from renewable sources, their high-cost relative to conventional petroleum-based solvents limits their industrial scalability.

Ethyl lactate, derived from renewable biomass, is among the least expensive green solvents, costing approximately £38/kg (data sourced from Merck). However, its applicability is restricted, as it is primarily effective for dissolving CA and PLA. More versatile green solvents, such as Cyrene and GVL, have gained attention in membrane research due to their compatibility with a wider range of polymers. Nevertheless, Cyrene is almost twice as costly as traditional solvents like NMP and DMF, while GVL is nearly five times more expensive, posing a considerable economic challenge.

Additionally, emerging green solvents such as [EMIM]­[OAc] and methyl lactate are significantly more costly, exceeding £18,000/kg and £2000/kg, respectively (data sourced from Merck). Leading from that, the high cost made them impractical for routine use in large-scale membrane fabrication.

### 2.4 Biopolymers as Green Alternatives to Fossil-derived Polymers

Biopolymers are polymers derived from living organisms such as microorganisms, plants, or animals and are composed of biodegradable, renewable monomers. Unlike synthetic polymers, biopolymers are inherently sustainable and environmentally friendly, making them an attractive alternative to petroleum-based polymers. In recent years, their potential as a sustainable material has garnered significant attention in membrane fabrication, particularly as the membrane market and research community shift toward addressing global challenges such as plastic waste accumulation and resource depletion.^8,101^ Biopolymers exhibit unique characteristics, including biodegradability and versatility in material design, which positions them as a promising class of materials for sustainable applications. Broadly, biopolymers can be categorized into three main types: polysaccharides, polyesters, and proteins. This section highlights recent advancements in membrane fabrication using biopolymers, with a focus on membrane structure, material characteristics, and filtration performance.

#### 2.4.1 Polysaccharides

Polysaccharides are long-chain polymeric carbohydrates composed of monosaccharide units linked by glycosidic bonds. Figure 6 illustrates the various types of polysaccharides with their sources. These polymers often occur in heterogeneous forms, with slight modifications in their repeating units. Depending on their structure and composition, polysaccharides exhibit diverse properties that differ significantly from their monosaccharide building blocks, including variations in crystallinity, solubility, and mechanical behavior. Certain polysaccharides are amorphous, while others are water-insoluble, offering a wide range of functionalities for membrane applications.

**Figure 6:**
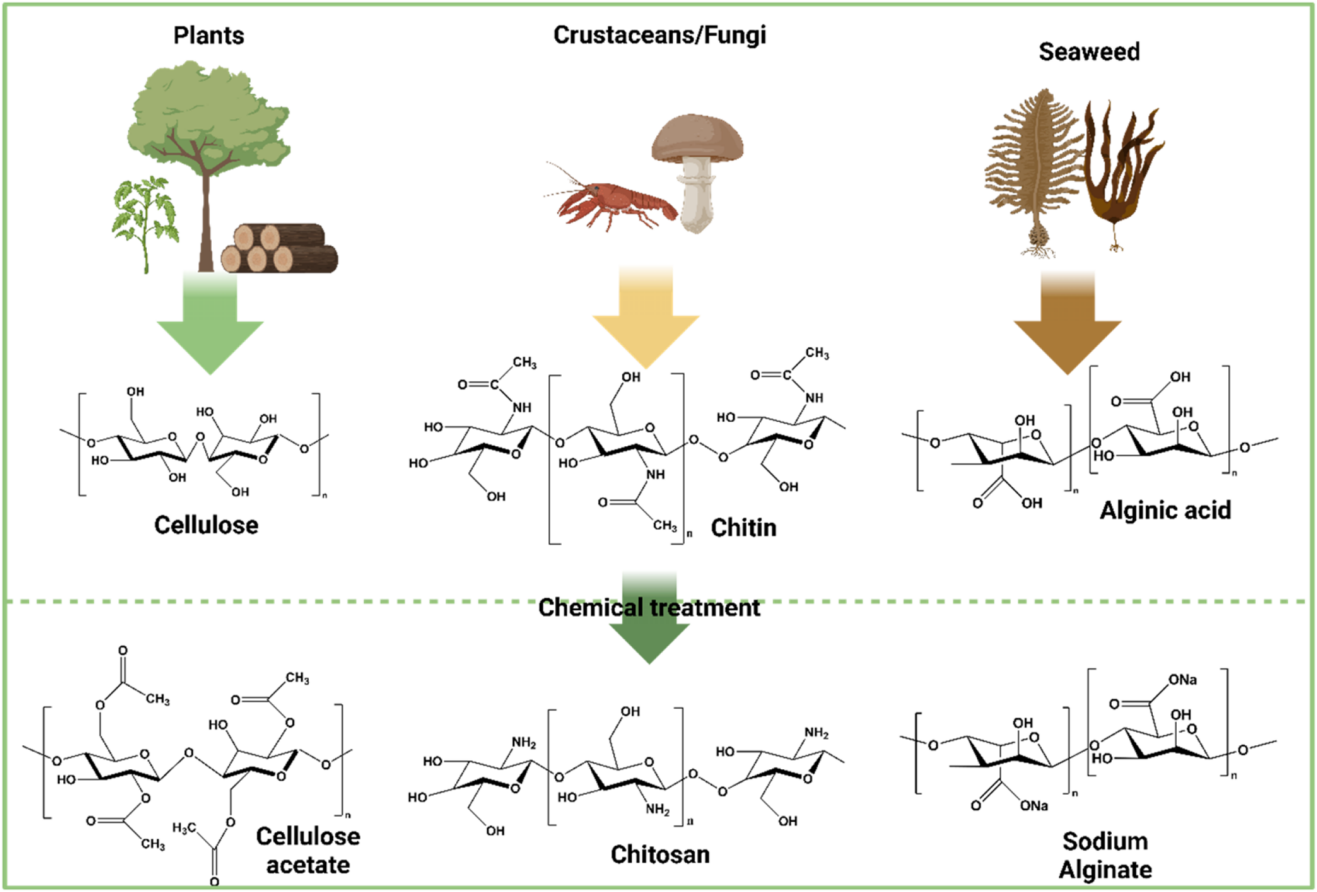
Schematic diagram of polysaccharide-based membrane materials derived from various natural sources, including plants, crustaceans, fungi, and algae. The diagram depicts the extraction and processing of biopolymers such as cellulose, chitin, and alginate from these sources, which are then combined into a unified polysaccharide structure for membrane fabrication, emphasizing a sustainable and biobased approach to material design for potential applications in wastewater treatment.

##### 2.4.1.1 Cellulose-based Derivatives

Cellulose is a polysaccharide predominantly found in plants or produced via the microbial fermentation of sugars, making it one of the most abundant biopolymers in nature. Its structure consists of long macromolecular chains of β-d-glucose units. Due to its abundance of hydroxyl groups, cellulose exhibits exceptional hydrophilicity—an essential property for water treatment membranes. However, pristine cellulose is rarely utilized in membrane fabrication because of its low solubility in most solvents and its limited mechanical and thermal stability.^8^ To overcome these limitations, various cellulose derivatives can be synthesized through chemical modifications. For example, CA is produced by treating cellulose with acetic acid, acetic anhydride, and sulfuric acid as a catalyst,^102^ while hydroxyethyl cellulose (HEC) is obtained by reacting ethylene oxide with alkali-treated cellulose.^103^

Although pristine cellulose is not widely applied in membrane fabrication, some progress has been made in dissolving cellulose. Li’s group developed a cellulose-based NF membrane using the layer-by-layer (LbL) technique for sodium chloride (NaCl) removal.^104^ Bamboo cellulose and chitosan were dissolved in *N*-methylmorpholine-N-oxide (NMMO) solvent and processed into cellulose support membranes using the immersion gel method. Chitosan introduced positive charges into the support matrix, while sodium carboxymethyl cellulose, an anionic cellulose ether, was applied as a top-coating layer. The resulting composite membrane demonstrated a rejection rate of 36% and a flux of 121 L/m^2^·h for 500 ppm of NaCl at 0.3 MPa, showcasing a scalable and convenient method for NF membrane production.

CA, an ester derivative of cellulose, is one of the most frequently used cellulose-based materials in membrane fabrication. Recently, Ananthi et al. fabricated a CA-based UF membrane incorporating MOFs for the removal of micropollutants (Figure 7a).^105^ Using NIPS method, CA, polystyrene, and MIL-88A (a MOF composed of Fe clusters and fumarate linkers) were dissolved in an acetone/chloroform mixture and cast into a UF membrane. The fabricated membrane exhibited excellent separation performance, achieving flux values of 75.2 L/m^2^·h with an 80% removal rate for diclofenac and 86.9 L/m^2^·h with a 78% removal rate for ciprofloxacin.

**Figure 7:**
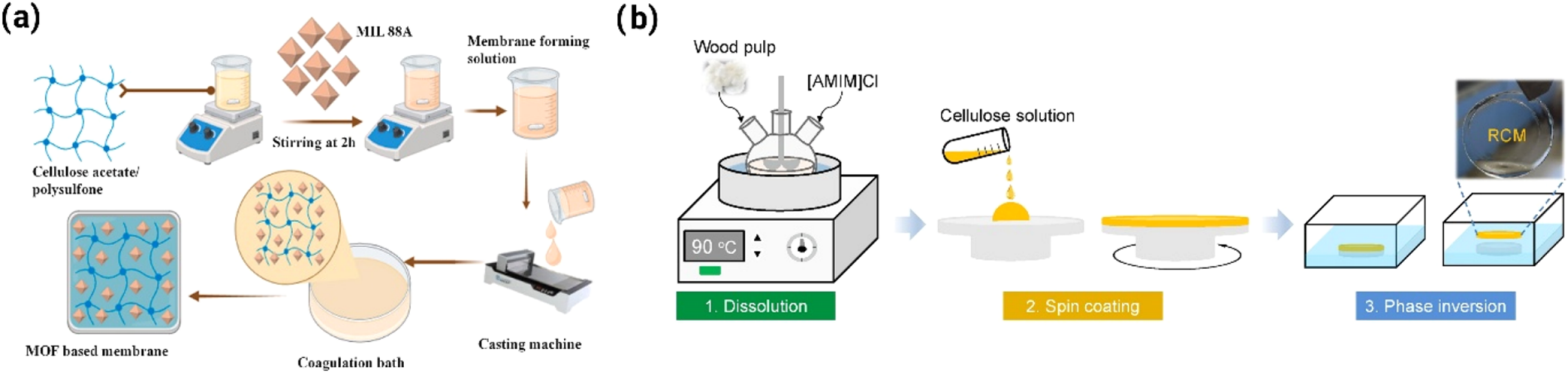
Schematic diagram of (a) CA/MOF/PSf membrane fabrication via NIPS^105^ and (b) regenerated cellulose membrane fabrication via spin-coating coupled with NIPS.^108^

Modified CA membranes have also been developed for specific applications. Ning et al. prepared deacetylated cellulose acetate (d-CA) nanofibers via electrospinning for oily water purification.^106^ CA membranes were treated with a NaOH mixture to produce d-CA membranes, which were then reinforced with bacterial cellulose for improved performance. The resultant membranes demonstrated remarkable hydrophilicity (water contact angle >40°) and achieved exceptional separation performance in *n*-hexane/water emulsions, with permeance values of 5479 L/m^2^·h·bar and 99.4% removal efficiency (with emulsifier) and 9364 L/m^2^·h·bar with 98.3% removal efficiency (without emulsifier).

To achieve sustainability, recycling cellulose-based materials is another promising approach. Torkashvand et al. fabricated CA membranes for forward osmosis (FO) applications using recycled cigarette butts as the source material.^107^ Cigarette filters, which consist of CA, were manually separated, purified, dissolved in NMP, and cast using the NIPS method. The recycled membranes demonstrated effective heavy metal removal, achieving a flux of 13.2 L/m^2^·h and removal efficiencies of 85–90% for chromium­(IV), cadmium, and lead ions.

Regenerated cellulose (RC), another cellulose subclass, involves converting natural cellulose into soluble derivatives through chemical treatment. Song et al. employed RC to fabricate membranes for OSN (Figure 7b).^108^ The RC was sourced from a processed wood pulp board from a paper mill. Using a green ionic liquid, 1-allyl-3-methylimidazolium chloride ([AMIM]­Cl), RC was dissolved and fabricated into membranes via spin coating and NIPS. The membranes, with thicknesses ranging from 150 to 350 μm, exhibited ethanol permeance of 30 L/m^2^·h·bar and high molecular selectivity for Alcian blue/Rifampicin (294) and Alcian blue/Tetracycline (68) mixtures, showcasing their potential for OSN applications.

##### 2.4.1.2 Chitosan

Chitosan is a biopolymer composed of linear amino polysaccharides consisting of β-d-glucosamine and *N*-acetyl glucosamine units. Typically, it appears as a white, hard, and inelastic material. Chitosan is derived from the alkaline deacetylation of chitin, which is abundant in the exoskeletons of crustaceans and the cell walls of fungi. Its biodegradability, biocompatibility, and nontoxic nature make it a promising candidate for membrane fabrication.^109,110^

Chitosan’s inherent hydrophilicity, attributed to its abundance of amine and hydroxyl functional groups, enhances its adsorption capabilities for charged contaminants. For instance, Wu et al. developed a chitosan-based cellulosic nanofibril membrane composite for dye adsorption.^111^ Anionic cellulose nanofibrils were incorporated to improve the acid resistance of the cationic chitosan via ionic and hydrogen bonding. The composite membrane was fabricated using a gel-casting method. The resulting chitosan/cellulose composite exhibited a high adsorption capacity for methylene blue, a positively charged dye, achieving an adsorption rate of 14.7 mg/g under strongly acidic conditions. Furthermore, the membrane demonstrated excellent reusability, maintaining an 85% desorption efficiency after multiple adsorption–desorption cycles.

In addition to dye removal, chitosan has been applied in nuclear wastewater decontamination. A chitosan-based thin-film composite NF membrane was synthesized via interfacial polymerization (IP) followed by polyethylenimine (PEI) postfunctionalization.^112^ In this study, chitosan was cross-linked with trimesoyl chloride to form a PA selective layer, and surface functionalization with PEI introduced cationic functional groups. The fabricated membrane exhibited high water permeance (17.4 L/m^2^·h·bar) and excellent rejection rates for CoCl_2_ (94.8%), SrCl_2_ (93.1%), and CsCl_2_(32.3%).

##### 2.4.1.3 Alginate

Alginate is a seaweed-derived, hydrophilic polysaccharide consisting of alternating blocks of β-d-mannuronic acid (M) and α-l-guluronic acid (G) residues arranged in homopolymeric (MM or GG) or heteropolymeric (MG) sequences. Alginates are widely used due to their biocompatibility and gel-forming properties. Xia et al. utilized sodium alginate (SA) as a hydrogel membrane coupled with Cu^2+^ metal ion for surface modification to the RO thin film composite (TFC) via LbL method.^113^ SA is a natural polyelectrolyte with high density of hydroxyl group and carboxyl groups, making it easy to form ionic bonds with metal ions to form polyelectrolyte hydrogel. The surface-modified RO membrane possesses water flux up to 71.4 L/m^2^·h, which is 93% better than the unmodified membranes without any significant decrease in salt rejection. Furthermore, the flux recovery rate of the surface-modified membrane is 26% better than that of the unmodified membrane using bovine serum albumin (BSA) as the modeled fouling tests. The excellent antifouling capabilities of the membrane can be attributed to the synergy of the polyelectrolyte hydrogel cross-linked with metal ions.

Due to the excellent hydrophilicity of alginate compounds, they were extensively used in oil/water separation. Wang’s group prepared a SA electrospun membrane incorporated with tannic acid (TA) to form TA-Fe­(III) complexes via a coordination reaction.^114^ Then a layer of β-FeOOH nanoparticles was constructed on the surface of the membrane. The resulting nanofibrous membrane exerts excellent underwater superoleophobicity and anticrude oil fouling properties. The nanofibrous membrane has high flux (∼1900 L/m^2^·h) and separation efficiency (99.6%) for crude oil-in-water emulsions. Interestingly, the membrane also possesses photo-Fenton self-cleaning that can degrade the absorbed pollutants on the membrane surface.

#### 2.4.2 Biopolyesters

Shifting from polysaccharide-based solutions, biopolyesters offer distinct advantages and challenges in membrane design. Polyesters are a class of polymers characterized by one or more repeated ester linkages in their main molecular backbone, and they are most commonly associated with synthetic fibers. In contrast, biopolyesters are a subclass of polyesters that are biodegradable and biocompatible, derived from renewable biological sources. Biopolyesters have garnered significant attention as sustainable substitutes for petroleum-based polymers. They can be synthesized through various methods, including microbial fermentation,^115,116^ chemical synthesis using biobased monomers^116^ or direct extraction from natural sources.

##### 2.4.2.1 Polylactic Acid

Polylactic acid (PLA) is a biopolymer composed of lactic acid monomers, where the lactic acid is typically derived from the fermentation of sugars or starches.^116^ Among many biopolymers, PLA stands out as a promising material for membrane fabrication due to its processability, low environmental impact, high mechanical and thermal strength, and biocompatibility.^117–119^

Despite its potential as a sustainable alternative to conventional polymers, PLA is intrinsically hydrophobic, which limits its applicability in water treatment. To address this limitation, a PLA-based hollow fiber membrane was synthesized using the NIPS method, with a surfactant (Tween-80) and poly­(vinylpyrrolidone) (PVP) as coadditives to enhance the hydrophilicity of the membrane surface.^120^ The modified PLA membrane exhibited a significant improvement in water permeance, increasing by 816.4% (151.2 L/m^2^·h·bar) compared to pristine PLA membranes. Tween-80 improved the dispersity of PVP, facilitating its migration toward the membrane surface via hydrogen bonding, which altered the surface properties and pore structure.

Given the challenges posed by membrane fouling, recent studies have focused on incorporating antifouling mechanisms into membrane fabrication. For instance, Khalil et al. developed an asymmetrical UF PLA membrane for the removal of organic substances from wastewater.^121^ Using the NIPS method, the fabricated membrane achieved a high separation efficiency of 92% for organic contaminants and demonstrated excellent antifouling properties, with a flux recovery ratio (FRR) of 89%.

In recent years, adsorptive membranes have gained prominence due to the integration of adsorption processes with membrane technology. Nassar et al. fabricated an adsorptive PLA-based membrane incorporating positively charged carbon nanotubes/graphene oxide nanohybrids via the NIPS method.^122^ This membrane was applied for the adsorptive removal of nitrogen and phosphorus nutrients from wastewater, achieving separation efficiencies of 90.1% for ammonium-nitrogen and 71.3% for phosphate ions in raw municipal wastewater. Moreover, the membrane demonstrated excellent regenerative ability, maintaining stability and reusability after simple washing with water, underscoring its potential for real-world wastewater applications.

##### 2.4.2.2 Polyhydroxyalkanoate

Similar to PLA, polyhydroxyalkanoates (PHAs) are a family of biodegradable polyesters naturally produced by microorganisms as intracellular energy and carbon storage materials. PHAs can be categorized into three groups based on the carbon chain length of the hydroxylalkanoate (HA) unit: short-chain length (scl-PHAs, fewer than six carbons), medium-chain length (mcl-PHAs, six to 14 carbons), and long-chain length (lcl-PHAs, 14 or more carbons).^123^ The molecular structure and properties of PHAs vary depending on factors such as synthesis conditions, the type of carbon source fed to microorganisms, and the specific microorganisms employed.^124–126^ The most common and widely used PHAs are polyhydroxybutyrate (PHB) and poly­(3-hydroxybutyrate-*co*-3-hydroxyvalerate) (PHBV) in membrane fabrication, though over 150 types of PHAs have been synthesized to date.

MF membranes made of PHAs can also perform well in filtration applications compared to conventional polymers. Tomietto et al. fabricated an MF membrane using PHBV via the NIPS method.^127^ They also added hydrophilic additives such as ethylene glycol and poly­(ethylene glycol) into the fabrication process, altering the morphology of the membrane structure. Due to the increased pore size, the permeance was significantly increased by adding the additives at 480 L/m^2^·h·bar with decent clay dispersion rejection.

An environmentally friendly PHB-based biodegradable nanofibre coupled with SiO_2_ nanoparticles for gravity-driven oil–water separation was fabricated by Sariipek’s group.^128^ The nanofibers were prepared via electrospinning and they possess superhydrophobic properties, with water contact angle of 160.2°. Oil–water dispersion can be separated via gravity using the SiO_2_/PHB membrane, and the separation performance is excellent as well, with 99.4% removal efficiency and a permeance of 5000 L/m^2^·h.

##### 2.4.2.3 Poly­(butylene succinate)

Poly­(butylene succinate) (PBS) is an aliphatic biopolyester that can be synthesized via polycondensation of biobased succinic acid and 1,4-butanediol. The feedstock can be derived from renewable sources, such as corn or sugar cane. Although biobased PBS currently remains more expensive than fossil fuel-derived PBS, there is still some research revolving around the use of biobased PBS. Bang et al. prepared a nanofibrous membrane with biobased PBS using a solution blow spinning method, yielding 130 nm fiber diameters and high porosity (97.4%).^129^ The PBS nanofibrous membrane exerts high oil adsorption capacity (18.7–38.5 mg/g) and excellent separation efficiency for water/oil mixtures (99.4–99.98%) and emulsions (98.1–99.5%). In the degradation tests, the PBS membrane can be degraded either through hydrolysis or biodegradation processes.

#### 2.4.3 Proteins

##### 2.4.3.1 Silk Fibroin

Silk Fibroin (SF) is a natural protein polymer produced from the silk of silkworms () or certain spider species. It is composed of long polypeptide chains primarily made up of amino acids. In addition to its biocompatibility, SF is renowned for its excellent mechanical strength, high thermal stability, and strong performance in membrane-based water filtration applications.^130–132^ For dye removal, a biodegradable multilayered nanofibrous membrane incorporating lignin and SF was developed.^132^ The resultant lignin/SF membrane achieved a remarkable dye removal efficiency of 99.5% for contaminants such as crystal violet, methylene blue, and brilliant green via adsorption. Furthermore, the membrane demonstrated versatility by efficiently retaining metal ions such as copper and cadmium, highlighting its potential as a biobased membrane with minimal environmental impact.

SF is also an effective surface modifier in TFC membrane fabrication. Lee’s group utilized the abundance of hydroxyl groups in SF to coat PA TFC membranes.^133^ The SF-coated PA membranes exhibited a smaller surface ζ-potential compared to pristine PA membranes, which reduced carboxyl group scaling on the surface. Additionally, the SF-coated membranes demonstrated exceptional antifouling properties, including five times lower irreversible scaling resistance, 50% higher FRR, and a filtration duration four times longer than unmodified membranes.

##### 2.4.3.2 Collagen

Collagen is a structural protein abundantly found in the human body, accounting for approximately 25–35% of total protein content. It is widely employed as a biomedical material due to its biodegradability, biocompatibility, and low immunogenicity.^134,135^ Collagen is commonly extracted from animal byproducts, with its molecular structure comprising three twisted polypeptide α-chains that form a triple helix.^136^

The molecular structure of collagen contains numerous hydrophilic groups (−OH, −COOH, –NH_2_), making it a promising material for membrane fabrication. Desiriani et al. incorporated collagen and polyphenols from green tea into a polymeric membrane matrix to enhance the antifouling and antibacterial properties of PES membranes.^135^ The addition of 1 wt % collagen and 3 wt % polyphenols significantly improved membrane performance compared to the pristine PES membrane. The modified membrane achieved a pure water flux of 359.9 L/m^2^·h, an FRR of 85.1% for BSA solutions, and a 93.5% removal efficiency for . The incorporation of polyphenols improved the membrane’s antibiofouling properties by providing antibacterial effects, while collagen enhanced antifouling performance through its intrinsic hydrophilicity. The combined effects of these additives resulted in improved water uptake, porosity, hydrophilicity, solute rejection, and flux recovery, demonstrating the potential of collagen-polyphenol-modified membranes for practical water treatment applications.

#### 2.4.4 Economic Viability of Biopolymers

Biopolymers present a sustainable alternative to conventional polymers for membrane manufacturing, offering environmental benefits due to their biodegradability. However, their economic viability is constrained by high production costs, limited feedstock options, and challenges in waste management.

As compared to conventional polymers, biopolymers have higher production costs and poses as a major challenge toward the widespread use of it. For instance, conventional polymers costs around $1000–1500/ton, while the most commonly used polymer, PLA, cost around $4,000/ton and can reach as high as $15,000/ton for PHA.^137^ It also has been reported that biopolymers are 7.5 times more expensive than conventional petroleum-derived products.^137^ Even the biobased ($5/kg-$6/kg) and petroleum-based ($4/kg-$4.5/kg) PBS have a slight discrepancy in their cost, leading to a more favored economic position for the latter product.^138^

Feedstock availability and type play a critical role in determining the cost and sustainability of biopolymer production. There are currently three generations of carbon sources for biopolymer production and synthesis. first generation feedstocks, derived from carbohydrate-rich food crops like maize and sugar cane, are widely used but compete with human food supplies and require significant land, raising concerns about food security and environmental impact. second generation feedstocks, sourced from nonfood biomass such as bagasse, husks, and bone waste, repurpose agricultural residues, reducing competition with food resources and lowering production costs by minimizing cultivation needs. third generation feedstocks, based on algae, offer high biomass productivity due to efficient CO_2_ uptake and can be cultivated in nonarable environments, enhancing yield and sustainability. Current research prioritises second- and third-generation feedstocks to improve economic and environmental outcomes.^137^ By repurposing waste, second-generation feedstocks enhance the economic value of biopolymers, while algae-based third-generation sources promise higher yields with minimal resource inputs. Transitioning to these feedstocks could lift up the cost barrier and improve the scalability of biopolymer production.^139^

Biopolymers are known for their biodegradability, which simplifies EoL waste management compared to conventional polymers. However, the degradation process varies significantly depending on environmental conditions and biopolymer type. For instance, PLA can decompose into its constituent parts within three months in a controlled composting environment with adequate heat, moisture, and microbial activity. In contrast, under natural conditions lacking excessive light and oxygen, a PLA bottle may persist for 100 to 1000 years.^137^ This variability complicates waste management and increases disposal costs, particularly for membrane applications where biopolymer dismantling from modules would need additional expenses.

### 2.5 Integration of Sustainable Approaches in Membrane Fabrication

To maximize the sustainability of polymeric membrane fabrication, the integration of mechanosynthesis, green solvents and biopolymers offers a synergistic pathway to reduce environmental impact while maintaining high performance. By combining this approach, it is possible to create membranes that leverages the solvent-free synthesis of advanced materials, the low toxicity profile of green solvents and the renewability of biopolymers. This integrated framework aligns with the principles of green chemistry and supports the closed-loop membrane production system, as illustrated in Figure 1.

One promising and straightforward approach is the use of mechanosynthesized materials, such as MOFs, as fillers or building blocks in biopolymer-based membranes fabricated with green solvents (Figure 8). For instance, mechanochemically synthesized water stable UiO-66 can be incorporated into CA membranes using ethyl lactate as a solvent via NIPS. The high surface area, tunable porosity and diversified derivatives of UiO-66 enhance the membrane’s permeance and selectivity for contaminants like dyes or micropollutants, while ethyl lactate ensures a low-toxicity fabrication process, and CA provides a renewable polymer matrix. This combination could yield membranes with enhanced separation efficiency and reduced environmental footprint. Recently, a mixed matrix membrane consisting CA and various MOFs was fabricated using dimethyl carbonate as the green solvent for gas separation application.^140^ This study sheds light to the potential of incorporating mechanosynthesized MOFs or COFs into the polymer matrix as a water separation membrane, which was discussed earlier.

**Figure 8:**
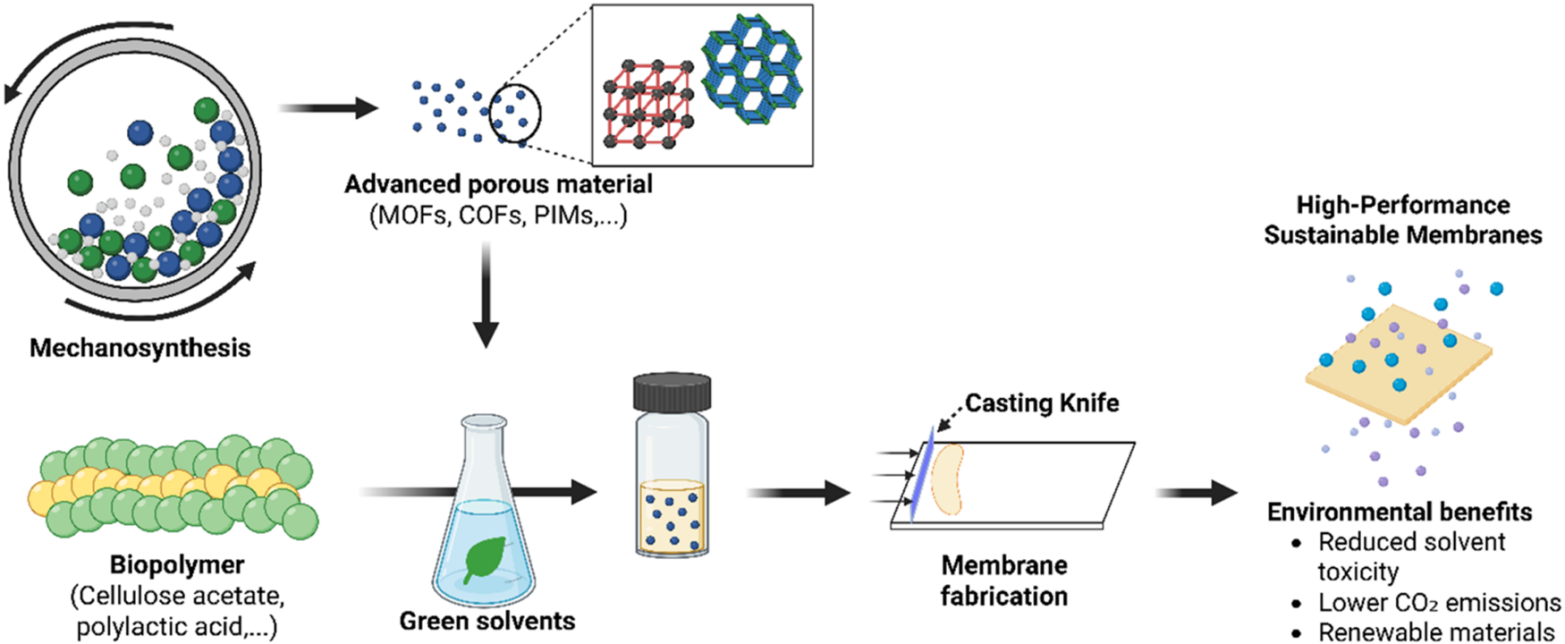
Schematic diagram illustrating the integration of mechanosynthesis (e.g., MOFs, COFs, PIMs), green solvents (e.g., Cyrene, GVL), and biopolymers (e.g., CA, PLA) in the fabrication of high-performance membranes via processes like NIPS.

Although the integration of the sustainable practices may seem ideal, some challenges persist in the adoption of this integration. Challenges in integration include ensuring compatibility between mechanosynthesized materials and green solvents, as well as optimizing biopolymer stability for high-pressure applications, such as NF/RO. Additionally, green solvents could be explored for dissolving mechanosynthesized COFs to form composite membranes with enhanced selectivity.

### 2.6 Policy and Regulatory Framework for Sustainable Membranes Manufacturing

A successful transition to sustainable membrane manufacturing requires a robust policy and regulatory framework that supports the adoption of green chemistry and circular economy principles. These frameworks, comprising chemical regulations, environmental legislation, voluntary standards, and funding incentives, create an enabling environment for the integration of sustainable practices, as discussed in previous sections, while also facilitating alignment with circular economy objectives explored later in the next section.

Chemical regulations are instrumental in driving the shift away from hazardous substances toward more environmentally benign alternatives. Within the European Union, the REACH regulation restricts the use of toxic solvents such as NMP and DMF, thereby encouraging the adoption of greener alternatives including biobased solvents such as Cyrene and GVL, as previously highlighted.^141^ In the United States, the Environmental Protection Agency (EPA) enforces the TSCA, which regulates the production and use of chemicals across various industries, including membrane manufacturing.^142^ Additionally, the US Clean Water Act mandates the treatment of solvent-contaminated effluents, thereby promoting the uptake of solvent-free and wastewater recycling technologies.^143^ Consequently, these regulatory measures foster the use of biobased solvents and innovative synthesis approaches such as mechanosynthesis, helping to reduce the environmental footprint of membrane production.

In addition to legislative instruments, voluntary standards and strategic funding mechanisms play a vital role in advancing sustainable manufacturing. International standards such as ISO 14001 provide a framework for environmental management systems, guiding organisations toward more resource-efficient and environmentally responsible operations.^144^ Furthermore, funding initiatives such as Horizon Europe and the UK’s Engineering and Physical Sciences Research Council (EPSRC) have significantly supported research into green chemistry, including the development of biopolymers and sustainable solvents for membrane fabrication.

The incorporation of circular economy principles, which will be discussed in the next major section, is also a major driving force for sustainable membrane production. Policies such as the European Union’s Circular Economy Action Plan promote material recovery and recycling strategies, which are directly relevant to the advancement of EoL membrane recycling.^145^ These measures contribute to waste reduction and improve material reusability, aligning with the SDG 12 on responsible consumption and production.

In summary, a comprehensive and forward-looking policy and regulatory landscape, characterized by stringent legislation, voluntary compliance standards, and targeted financial support, is essential to fostering innovation and accelerating the transition to sustainable membrane manufacturing in line with global environmental and socio-economic priorities.

## 3 Polymeric Membrane for Circular Economy: Downcycling, Upcycling and Repreparation

To ensure the long-term viability of membrane technology, it is crucial to evaluate the sustainability of both the fabrication processes and the management of EoL membranes. Synthetic polymeric membranes are commonly classified into four categories based on pore size: MF, UF, NF, and RO, with pore size decreasing progressively across these types. Despite their efficacy, all synthetic membranes inevitably reach the end of their functional lifespan, compelling the development of sustainable solutions for their disposal or recycling. Landaburu-Aguirre et al. estimated that by 2025, nearly 30,000 tonnes of discarded RO membranes would accumulate in landfills worldwide.^146^ Additionally, regional differences in membrane management practices can result in significantly shorter operational lifespans for membranes compared to reported averages, worsening the problem of membrane waste generation.^147^

A circular economy framework, which emphasizes closing material loops to prevent waste and minimize environmental impact, offering a promising pathway for addressing the sustainability challenges associated with membrane technology. Recycling EoL membranes plays a critical role in advancing a circular economy by extending the functional lifespan of membranes and reducing waste. Membrane recycling can generally be categorized into three types: upcycling, downcycling, and repreparation. Upcycling involves converting EoL membranes into materials of higher quality than the original, while downcycling produces materials of lower quality. On the other hand, repreparation restores EoL membranes to a state comparable to that of pristine membranes. In this section, we highlight the trend of downcycling, upcycling, and the repreparation of EoL membranes. These circular strategies collectively pave the way for sustainable membrane lifecycles, setting the stage for future challenges and opportunities.

### 3.1 Downcycling

Membrane downcycling represents a promising recycling strategy for EoL RO membranes, enabling their transformation into lower-pressure filtration systems, such as NF, UF, or MF membranes. This approach typically employs sodium hypochlorite (NaClO) treatment to selectively degrade or remove the PA selective layer, which is the primary functional component of RO membranes (Figure 9a).^148^ The oxidative action of NaClO disrupts the molecular structure of the PA layer, facilitating its controlled removal and allowing the underlying support structure to be repurposed for less selective filtration applications.^149^ Alternative oxidative agents, such as hydrogen peroxide (H_2_O_2_) and potassium permanganate (KMnO_4_), have also been explored for PA layer removal.^150^ However, these compounds often generate oxidative residues that complicate downstream applications and increase the complexity of water treatment processes,^151^ making NaClO the preferred reagent for downcycling.

**Figure 9:**
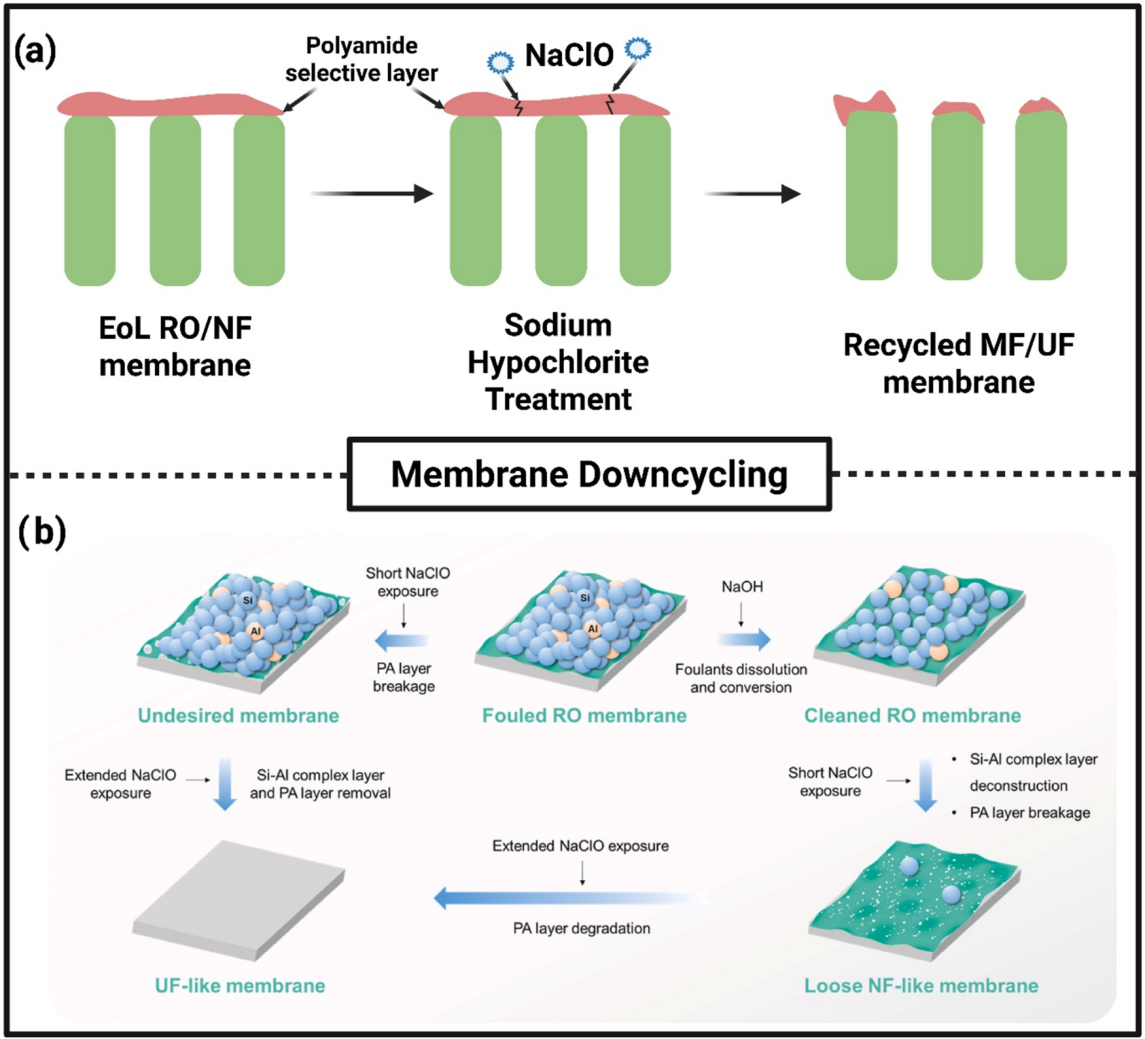
(a) Schematic diagram for a typical membrane downcycling process. (b) Downcycling of RO membrane to UF- or NF-like membranes using NaClO treatment for different treatment times. Reprinted from Wang et al.,^152^ Copyright 2024, with permission from Elsevier.

Recent advances in membrane downcycling have demonstrated its versatility and efficacy. For instance, RO membranes fouled with Si–Al complexes have been successfully converted into loose NF- or UF-like membranes through NaClO treatment (Figure 9b).^152^ Alkaline NaClO exposure gradually deconstructs the Si–Al fouling layer while simultaneously degrading the PA selective layer. Short chemical treatment results in a loose NF-like structure, whereas sufficient chlorine oxidation can fully eliminate the PA layer, yielding a UF-like membrane. Both resultant membranes exhibit satisfactory separation performance and enhanced antifouling properties, highlighting the adaptability of this method to fouled EoL membranes.

Downcycled membranes have also shown potential for specialized applications, such as the removal of contaminants like uranium from groundwater to produce potable water.^153^ Two RO membranes were treated with varying NaClO concentrations to produce recycled NF-based membranes. A membrane exposed to 220,000 ppm·h of NaClO achieved a uranium removal efficiency of 71.6%. However, this membrane exhibited susceptibility to fouling, with a 53% reduction in flux from an initial value of 10.3 L/m^2^·h, underscoring a trade-off between selectivity and long-term performance that warrants further optimization.

An alternative downcycling strategy integrates cleaning, healing, and IP with NaClO treatment to enhance the functionality of recycled membranes.^154^ In this method, EoL RO membranes are first cleaned with NaClO to remove fouling, followed by complete PA layer removal to produce a UF-like support membrane. A healing step employing polyelectrolyte deposition via the LbL technique reduces surface hydrophobicity, facilitating subsequent IP to reintroduce a PA layer. The resulting tight-NF membrane demonstrates exceptional performance, including a pure water permeance of 7.3 L/m^2^·h·bar and a Na_2_SO_4_ rejection rate of 99.5%, alongside improved long-term stability and fouling resistance. This multistep approach exemplifies how downcycling can not only repurpose EoL membranes but also restore or enhance their utility for high-efficiency separations.

### 3.2 Upcycling

Similar to membrane downcycling, the upcycling of EoL membranes represents a transformative approach to membrane recycling. This process involves reprocessing retired MF/UF membranes into higher-performance NF/RO membranes through the introduction of a PA selective layer via IP, thereby forming TFC membranes (Figure 10a).

**Figure 10:**
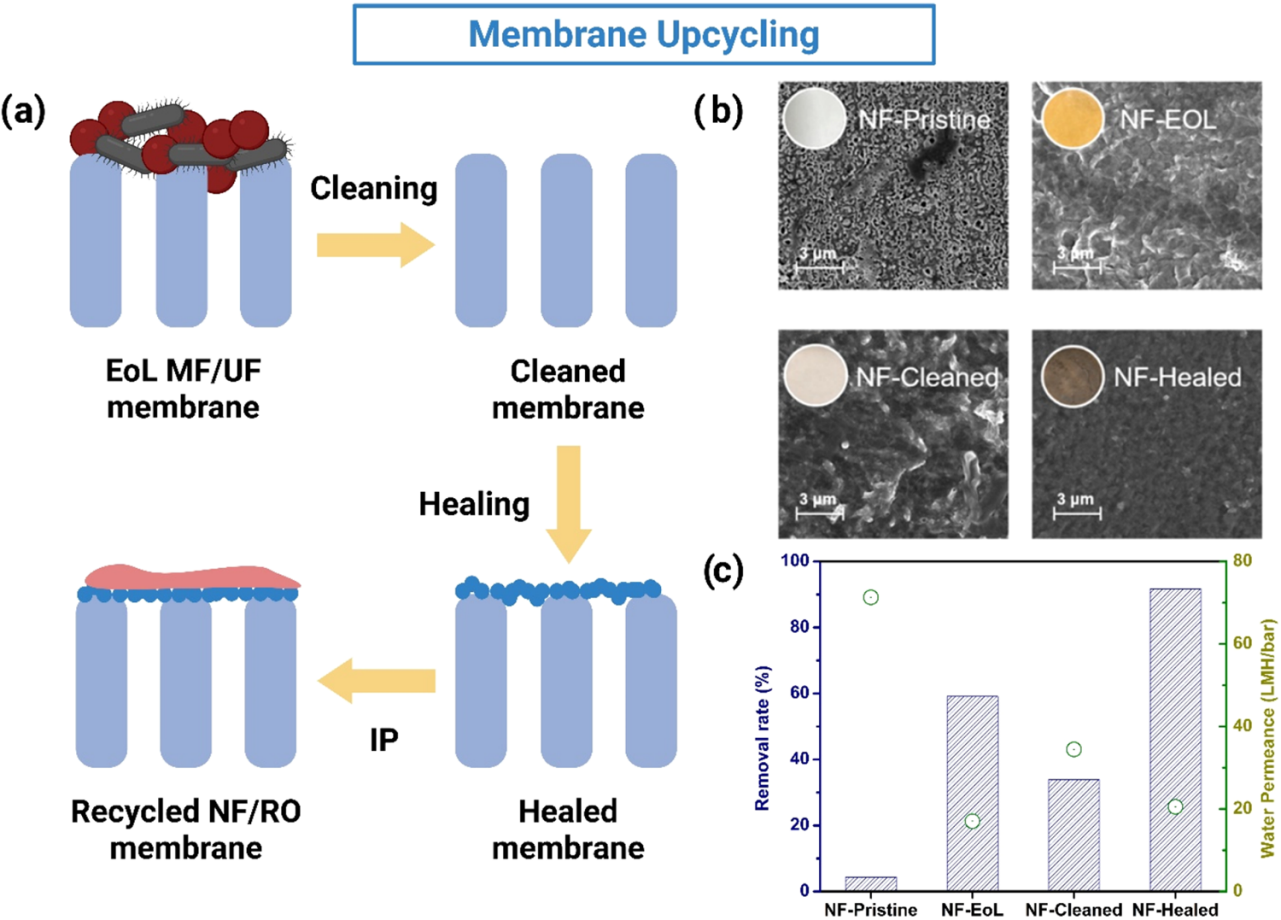
(a) Schematic diagram for cleaning-healing-IP method for membrane upcycling. (b) SEM diagram for the surface and (c) filtration performance of NF membrane made of pristine MF substrate, EoL MF substrate, cleaned EoL MF substrate and cleaned-healed EoL MF membrane. Reprinted with permission from Dai et al.^155^ Copyright 2021 American Chemical Society.

A widely adopted upcycling methodology follows a sequential cleaning-healing-IP protocol. The initial cleaning phase is critical for removing organic and inorganic foulants, while the subsequent healing step ensures uniform PA layer deposition by mitigating defects and preventing undesirable selective layer growth (Figure 10b).^155^ For instance, the efficacy of this approach was demonstrated through enhanced salt rejection performance: cleaned membranes exhibited ∼60% Na_2_SO_4_ rejection, whereas healed membranes modified with a hydrophilic polydopamine interlayer achieved 92.4% rejection (Figure 10c). Moreover, recent advancements have streamlined the healing process by substituting polydopamine with a tannic acid-Fe complex, reducing total processing time to <20 min while maintaining comparable separation performance.^156^ Economic analyses further underscore the viability of this approach, as the chemical costs for healing are offset by savings from avoided EoL membrane disposal and new membrane procurement.

Innovative strategies have also emerged that bypass traditional cleaning and healing steps.^157,158^ Biofouling residues on EoL MF membranes have been shown to regulate IP dynamics, facilitating controlled PA layer formation while simultaneously creating interfacial water channels between the selective layer and substrate.^157^ Additionally, surfactant incorporation during recycling has proven effective in regulating IP by enhancing surface wettability, thereby optimizing PA layer morphology and separation efficiency.^158^ Such simplified protocols not only reduce operational complexity but also improve cost-effectiveness, further advocating for membrane upcycling as a sustainable alternative.

These advancements highlight the technical and economic potential of membrane upcycling. By transforming EoL membranes into high-value TFC membranes through tailored chemical and structural modifications, this approach aligns with circular economy principles, offering a scalable and environmentally responsible solution for membrane lifecycle management.

### 3.3 Repreparation

Beyond conventional upcycling and downcycling, membrane repreparation offers a forward-looking pathway to align membrane EoL management with circular economy principles. This approach integrates sustainability into the membrane lifecycle by prioritising material reprocessability at the design stage. Repreparation involves dissolving retired polymeric membranes in organic solvents to form regenerated dopant solutions, which are subsequently reprocessed into new membranes with comparable or enhanced performance.

#### 3.3.1 Covalent Adaptable Network (CAN)

Covalent adaptable networks (CANs) have emerged as a groundbreaking class of materials for advancing recyclable membrane technologies.^159–164^ CANs are thermoset polymers engineered with dynamic covalent bonds, which undergo reversible cleavage or exchange reactions in response to external stimuli (e.g., heat, light, pH). This inherent dynamicity enables structural reconfiguration, self-healing, and closed-loop recycling—properties that address key challenges in membrane sustainability (Figure 11a).

**Figure 11:**
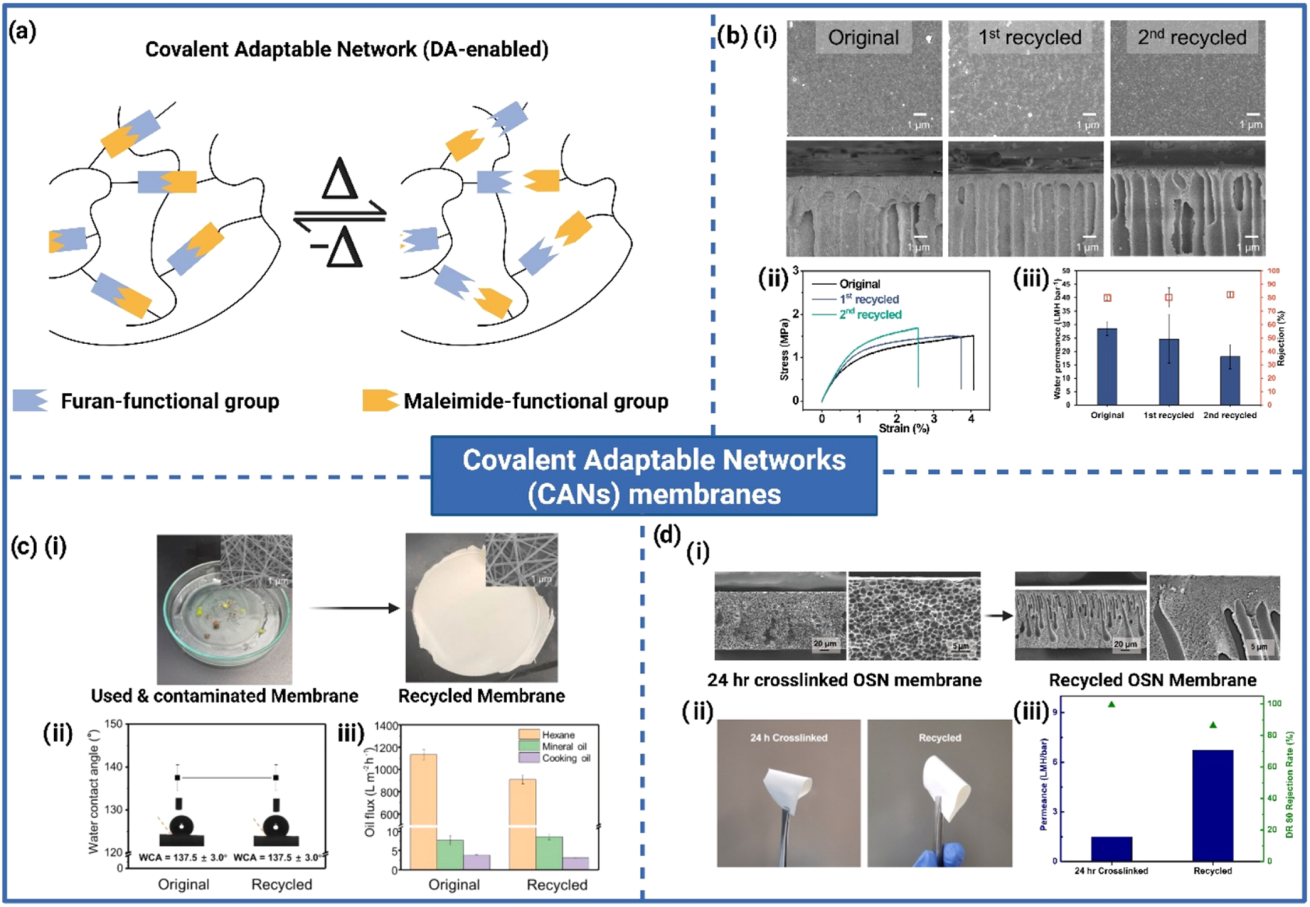
(a) Schematic diagram of the mechanism of bond-forming/breaking in CANs for Diels–Alder chemistry. (b) (i) The SEM diagram, (ii) mechanical properties, and (iii) filtration performance of the recyclable NF membrane. Copyright 2023 National Academy of Sciences. (c) (i) The captured image and SEM image, (ii) the water contact angle, and (iii) the oil flux of the recyclable electrospun membrane for oil/water filtration. Reprinted from Li et al.,^160^ Copyright 2020, with permission from Elsevier. (d) (i) The SEM image, (ii) the captured image for mechanical properties, and (iii) the filtration performance for the disulfide bonds enabled recycled membrane for OSN applications. Reprinted with permission from Ramírez-Martínez et al.^162^ Copyright 2024 American Chemical Society.

An innovative example is the Diels–Alder (DA) reaction, a thermally reversible dynamic covalent chemistry. In DA-based CANs, a conjugated diene reacts with a dienophile to form a six-membered cycloadduct, which can dissociate via retro-DA reactions under elevated temperatures. Leveraging this mechanism, Li et al. demonstrated the fabrication of a fully recyclable CAN membrane utilizing furan-maleimide DA adduct for water purification (Figure 11b).^159^ The pristine membrane exhibited robust separation performance, with a water permeance of 28.5 L/m^2^·h·bar and a molecular weight cutoff (MWCO) of 4581 g/mol, enabling efficient dye rejection.^159^ The pristine membrane exhibited robust separation performance, with a water permeance of 28.5 L/m^2^·h·bar and a molecular weight cutoff (MWCO) of 4581 g/mol, enabling efficient dye rejection. After using, the membrane was depolymerised at high temperature (90 °C) and reprocessed into new membranes via NIPS solvent-casting (Figure 11b­(i)). Remarkably, the recycled membranes retained their chemical integrity, mechanical strength (Figure 11b­(ii)), and filtration performance (Figure 11b­(iii)) over three reprocessing cycles, with no significant degradation in filtration efficiency. Moreover, the absorbed dye on the membrane before recycling has been removed using liquid extraction method, confirming that the fouled substances can be removed easily.

Electrospinning has emerged as a promising technique to integrate CANs into nanofibrous architectures, focusing on their dynamic covalent chemistry to enhance recyclability. For instance, CAN-based electrospun membranes exhibit exceptional hydrophobicity, high porosity, and robust chemical stability, enabling efficient oil/water separation with >99% oil removal efficiency (Figure 11c­(i)).^160^ The inherent recyclability of these membranes was demonstrated through thermal depolymerization via retro-Diels–Alder (retro-DA) reactions, which effectively disintegrated oil-fouling layers while preserving nanofiber integrity. Remarkably, reprocessed membranes retained their initial separation efficiency over two recycling cycles^160^ (Figure 11c­(ii,iii)), showing their durability in aggressive hydrocarbon environments. This approach not only addresses fouling challenges but also aligns with circular economy principles by minimizing material waste.

CANs have also been engineered for OSN, where solvent-resistant, recyclable membranes are critical for sustainable chemical processing. A notable example involves a furan-modified poly­(amide-imide)/bismaleimide DA system, which achieved an acetone permeance of 3.68 L·m^–2^·h^–1^·bar^–1^ and 95% rejection of Rose Bengal.^161^ While the original asymmetric membrane structure transitioned to a dense morphology postrecycling via solvent evaporation, the reprocessed membranes maintained mechanical integrity over three cycles, with negligible changes in tensile strength. However, the absence of postrecycling filtration data highlights a critical gap in current research.

Beyond thermally triggered systems, redox-responsive CANs leveraging disulfide dynamics offer a unique pathway for membrane recycling under mild conditions. Ramírez-Martínez et al. demonstrated this with a cysteamine-cross-linked poly­(ether imide) OSN membrane, which exhibited 99% rejection of Direct Red 80 in methanol (permeance: 1.4 L·m^–2^·h^–1^·bar^–1^) (Figure 11d).^162^ Chemical recycling was achieved via disulfide bond reduction using 1,4-dithiothreitol, which cleaved the polymer network while retaining the original mechanical strength postreprocessing (Figure 11d­(i)). Although dye rejection was decreased in recycled membranes, the retained solvent resistance and mechanical robustness emphasize the viability of redox-driven CANs for industrial OSN applications (Figure 11d­(ii,iii)). This approach bridges the gap between covalent adaptability and practical solvent stability, offering a blueprint for designing chemically resilient yet recyclable membranes. The absence of comprehensive filtration performance data postrecycling in CAN studies underscores the need for standardized testing protocols to validate their practical utility in desalination.

### 3.4 Economic Viability of Membrane Recycling

Membrane recycling offers significant environmental benefits, but its industrial adoption hinges on economic viability. Recent studies demonstrate that membrane recycling is not only sustainable but also highly cost-effective, making it an attractive option for water treatment industries. de Paula et al. highlight the eco-efficiency of membrane recycling, showing substantial cost savings alongside environmental benefits.^165^ Their analysis indicates that replacing 9000 UF membranes with recycled ones can yield annual savings of $10 million, representing a 98.9% cost reduction compared to purchasing new UF membranes. This dramatic cost advantage underscores the economic potential of membrane recycling. Further supporting these findings, another study reports a 27.1% cost reduction through closed-loop recycling of UF and NF membranes.^166^ The recycling process itself is cost-efficient, with associated costs estimated at only 0.7–2.1% of the total expenditure. These low recycling costs, combined with reduced expenses for membrane replacement and waste disposal, make recycling EoL membranes a financially superior alternative to procuring new ones. In summary, the economic advantages of membrane recycling, driven by significant cost reductions and minimal recycling expenses, position it as a compelling strategy for sustainable and cost-effective water treatment solutions.

## 4 Outlook and Perspective

This review highlights advancements in mechanosynthesis, green solvents, and circular economy integration for sustainable membrane fabrication. By comparing the data and information from the literatures, a comparative evaluation was illustrated to analyze the industrial adoption of green membrane fabrication technologies and the current conventional membrane fabrication methods (Table 4). While these innovations offer promising pathways to reduce environmental impact, several challenges must be addressed to enable their widespread adoption in industrial membrane manufacturing and desalination applications.

**Table 4:**
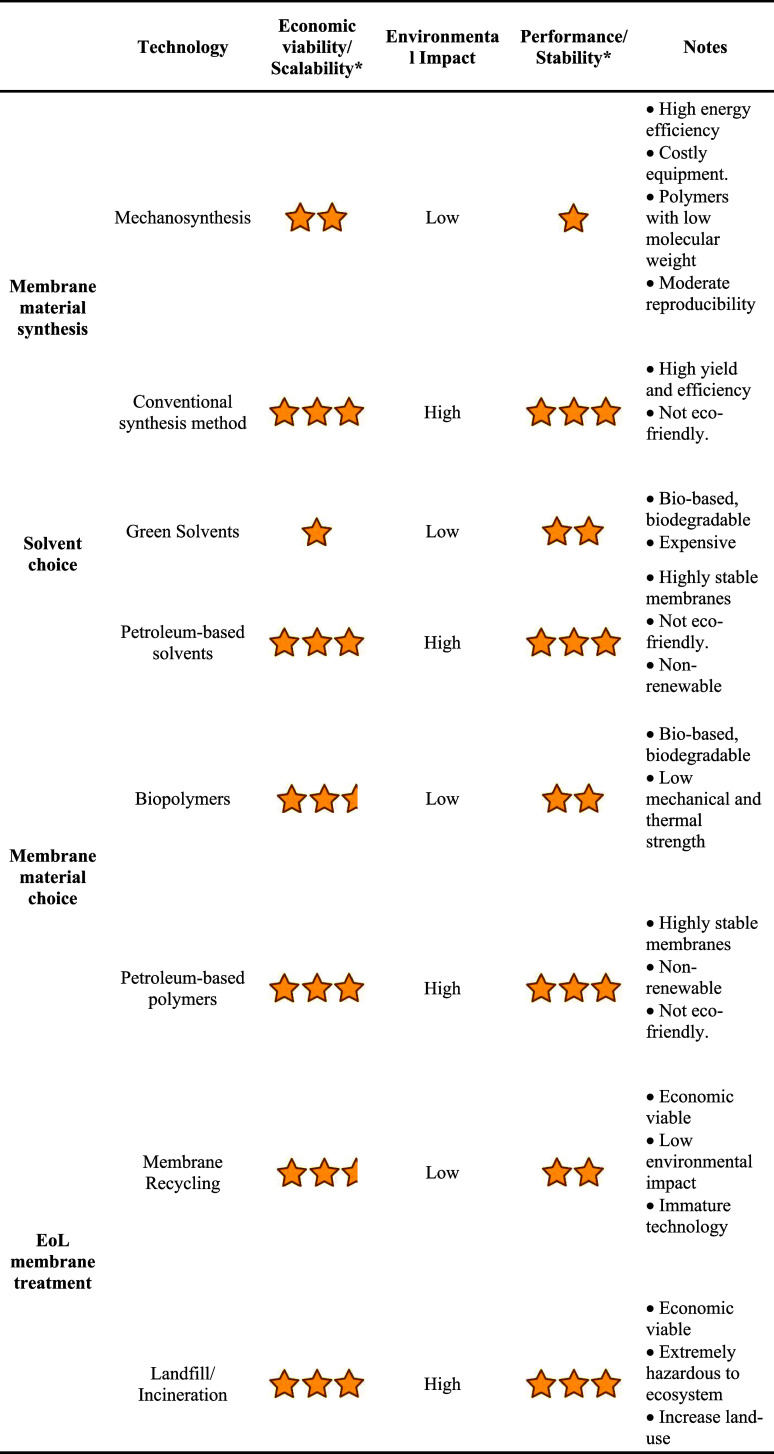
Comparative Evaluation of Sustainable Membrane Technologies and Conventional Membrane Fabrication Methods for Industrial Adoption

a Ratings are based on subjective evaluation of scalability, cost, and compatibility with existing industrial processes; (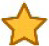:low, 
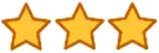
: high).

### 4.1 Challenges

Mechanochemical reactions provide energy-efficient, solvent-free routes for synthesizing advanced materials such as MOFs, COFs and PIMs. However, transitioning from lab-scale synthesis to industrial production remains a critical barrier. For instance, while twin-screw extrusion has enabled UiO-66-NH_2_ production at 1.4 kg/h,^24^ the high capital costs of specialized equipment (e.g., planetary mills, extruders) and the need for precise control over mechanical energy inputs hinder scalability. Furthermore, batch-to-batch variability in material properties (e.g., BET surface area, pore size distribution) may compromise membrane performance consistency in separation processes. Concurrently, green solvents, valued for their biodegradability and low toxicity, face compatibility challenges with conventional membrane polymers. For example, their application to PA-based TFC membranes remains underexplored. Moreover, membranes fabricated with green solvents frequently exhibit trade-offs between selectivity and permeance compared to those derived from traditional solvents (e.g., NMP, DMF),^70^ underscoring the need to balance sustainability with separation efficiency. Aside from that, biopolymers such as cellulose derivatives, PLA and PHAs present eco-friendly alternatives to petroleum-based polymers. However, their limited mechanical strength and thermal stability restrict their utility in high-pressure environments (e.g., RO- and NF-systems). For example, pristine cellulose suffers from poor solubility and durability,^8^ while PLA’s inherent hydrophobicity necessitates surface modifications to enhance water permeance, a process that increases manufacturing complexity and cost.

Sustainable materials and processes often incur higher costs than conventional counterparts. For instance, GVL (£368/kg, Sigma-Aldrich) and biobased PBS ($5/kg-$6/kg) are significantly more expensive than NMP (£82/kg, Sigma-Aldrich) and petroleum-based PBS ($4-$4.5/kg).^138^ Additionally, the capital investment required for mechanosynthesis infrastructure (e.g., twin-screw extruders) poses economic barriers to scaling. Without cost-competitive incentives, industrial adoption may lag, particularly in sectors prioritising operational efficiency. Further, circular economy strategies such as CANs depolymerization face practical challenges: industrial RO/NF modules, often encased in sealed shells, require costly dismantling and reprocessing steps for reuse.

While membrane recycling aligns with circular economy principles, performance degradation postrecycling remains a concern. Downcycled NF membranes exhibit reduced flux and increased fouling susceptibility,^153^ while upcycling processes demand multistep healing to restore filtration efficiency.^157^ Residual contaminants in upcycled membranes further limit their applicability in sensitive processes like desalination. Additionally, the lack of comprehensive data on the long-term stability and scalability of recycled CAN-based membranes raises uncertainties about their industrial viability. Furthermore, the increased logistical cost for membrane recycling could also be a potential economic issue to scale up the technology.

Aside from water treatment application, a critical challenge in advancing sustainable high-performance polymeric membranes for electrochemical and separation processes lies in addressing compatibility issues between polymers, green solvents, and operational environments. High-performance polymers, such as Nafion and s-PEEK, are prized for their thermal and chemical stability in applications like ion exchange membranes for flow batteries. However, transitioning to green solvents introduces compatibility challenges, as they can lead to undesired or suboptimal membrane morphologies. In electrochemical applications, membranes must withstand harsh electrolytes, where chemical degradation or fouling can compromise ionic conductivity and selectivity. These compatibility issues necessitate tailored solvent–polymer matching and surface modifications to ensure performance aligns with sustainability goals.

### 4.2 Future Potential

Convergence of advanced manufacturing, computational tools, and material science offers transformative opportunities for sustainable membrane technologies. Machine learning (ML) and artificial intelligence (AI) offer revolutionary potential for sustainable membrane manufacturing, addressing challenges in material selection, design and process optimization. For instance, graph neural networks (GNNs) can accelerate the discovery of biobased polymers and green solvents by predicting molecular properties, reducing reliance on toxic solvents.^167^ In addition, GNNs can also potentially model the degradation pathways of recyclable membranes, optimizing chemical processes for recycling strategies especially for vitrimer-based membranes. Besides GNNs, reinforcement learning (RL) and deep neural networks (DNNs) can streamline manufacturing processes, like mechanosynthesis and phase inversion processes, minimizing energy and solvent use. By integrating ML/AI with experimental and industrial data, researchers can overcome scalability barriers. Therefore, future efforts can be focused on curating comprehensive data sets and validating ML predictions to ensure practical implementation.

Biopolymers are promising sustainable alternatives for water treatment membranes but often exhibit insufficient mechanical strength for high-pressure applications. A facile strategy to address this limitation involves blending biopolymers with mechanically robust polymers (e.g., PVDF, PES, chitosan). Incorporating a small percentage of PVDF or PES into the biopolymer matrix can significantly enhance mechanical strength while preserving biodegradability. Additionally, the hydrogen bonding capacity of chitosan facilitates the formation of robust intermolecular interactions, yielding a stable membrane complex. Furthermore, reinforcing biopolymer matrices with nanomaterials (e.g., graphene oxide, carbon nanotubes) or developing hybrid organic–inorganic composites with inorganic materials (e.g., zeolites, MOFs) can substantially improve mechanical resilience, making them suitable for demanding applications like RO. Moreover, physical reinforcement through advanced fabrication techniques, such as electrospinning or LbL assembly, can further enhance the structural integrity of biopolymer membranes, optimizing their performance in water treatment processes.

Although membrane recycling presents a sustainable alternative to conventional EoL membrane disposal (landfill/incineration), it is often overlooked due to the logistical and operational challenges associated with transporting membranes to dedicated recycling facilities. These additional logistics not only increase costs but also contribute to the overall environmental footprint. To address these limitations, cost-effective circular economy strategies, such as on-site membrane upcycling or downcycling, or in situ CAN polymers repreparation, could revolutionize EoL membrane management. By localizing the recycling process, such approaches would streamline operations, reduce costs, and minimize environmental impacts, making sustainable membrane disposal more viable.

In addition to polymer-based membranes, inorganic materials such as ceramics, zeolites, and metal oxides have emerged as promising green alternatives for membrane applications. In contrast to their polymeric counterparts, inorganic membranes exhibit superior thermal and chemical resistance, along with enhanced mechanical strength and long-term durability. These properties significantly reduce the frequency of membrane replacement, particularly under harsh operating conditions involving aggressive cleaning agents such as NaClO. Despite these advantages, the fabrication of inorganic membranes often requires energy-intensive processes, such as sintering or pyrolysis, and may involve the use of volatile and hazardous chemicals through methods like chemical vapor deposition or sol–gel synthesis. These factors present environmental and safety challenges that must be addressed when evaluating the sustainability of inorganic membranes. Nevertheless, the successful commercialization of inorganic NF membranes, such as those developed by Inopor, demonstrates the growing viability of inorganic materials in the membrane technology market and their potential role in advancing sustainable separation processes.

To accelerate adoption, interdisciplinary collaboration among academia, industry, and policymakers will be essential to address technical bottlenecks, reduce costs, and establish standardized frameworks for sustainable membrane design and recycling.

By prioritising scalability, performance optimization, and lifecycle sustainability, next-generation membranes could achieve the dual objectives of environmental stewardship and industrial feasibility, which is critical for advancing green membrane technologies in a resource-constrained world.

## 5 Conclusions

This review underscores the progress in sustainable polymeric membranes through green chemistry and circular economy principles. Mechanosynthesis enables solvent-free synthesis of advanced materials, such as MOFs, COFs, and PIMs, reducing environmental impact. Green solvents, in particular, Cyrene and GVL offer low-toxicity alternatives for membrane fabrication, while biopolymers like cellulose derivatives and PLA provide renewable options, despite limitations in mechanical and thermal stability. These innovations collectively shift membrane technology toward sustainability.

Circular strategies such as downcycling, upcycling, and repreparation with CANs, extend membrane lifecycles, minimizing waste and conserving resources. However, challenges in scalability, economic viability, and performance persist, requiring further optimization. By advancing these eco-friendly approaches, sustainable membranes hold promise for water treatment and desalination, meeting industrial demands while prioritising environmental stewardship. Ongoing research and collaboration will be key to unlocking their full potential in a resource-limited future.

## References

[1] Biancalani, R. ; Hamouda, G. B. ; Marinelli, M. ; Chocholata, L. Progress on the Level of Water Stress – Mid-term Status of SDG Indicator 6.4.2 and Acceleration Needs, with Special Focus on Food Security, FAO 2024.

[2] UN-Water . Summary Progress Update 2021: SDG 6 — Water and Sanitation for All, UN-water 2021.

[3] Jones E., Qadir M., van Vliet M. T. H., Smakhtin V., Kang S.-m. (2019). The state
of desalination and brine production: A global outlook. Sci. Total Environ..

[4] Sholl D. S., Lively R. P. (2016). Seven chemical separations
to change the world. Nature.

[5] Yadav P., Ismail N., Essalhi M., Tysklind M., Athanassiadis D., Tavajohi N. (2021). Assessment of the environmental
impact of polymeric
membrane production. J. Membr. Sci..

[6] Razali M., Kim J. F., Attfield M., Budd P. M., Drioli E., Lee Y. M., Szekely G. (2015). Sustainable
wastewater treatment
and recycling in membrane manufacturing. Green
Chem..

[7] Nunes S. P., Culfaz-Emecen P. Z., Ramon G. Z., Visser T., Koops G. H., Jin W., Ulbricht M. (2020). Thinking the future of membranes: Perspectives for
advanced and new membrane materials and manufacturing processes. J. Membr. Sci..

[8] Galiano F., Briceño K., Marino T., Molino A., Christensen K. V., Figoli A. (2018). Advances in biopolymer-based membrane
preparation and
applications. J. Membr. Sci..

[9] Shanmugaratnam, T. ; Mazzucato, M. ; Okonjo-Iweala, N. ; Rockström, J. ; Ovink, H. The Economics of Water: Valuing the Hydrological Cycle as a Global Common Good; Global Commission on the Economics of Water, 2024.

[10] Baláž P., Achimovičová M., Baláž M., Billik P., Cherkezova-Zheleva Z., Criado J. M., Delogu F., Dutková E., Gaffet E., Gotor F. J. (2013). Hallmarks
of mechanochemistry: from nanoparticles to technology. Chem. Soc. Rev..

[11] Ardila-Fierro K. J., Hernández J. G. (2021). Sustainability
Assessment of Mechanochemistry by Using
the Twelve Principles of Green Chemistry. ChemSusChem.

[12] Friščić T., Childs S. L., Rizvi S. A. A., Jones W. (2009). The role of solvent
in mechanochemical and sonochemical cocrystal formation: a solubility-based
approach for predicting cocrystallisation outcome. CrystEngComm.

[13] Staudinger H., Leupold E. O. (1930). Über Isopren und Kautschuk, 18. Mitteil.: Viscositäts-Untersuchungen
an Balata. Ber. Dtsch. Chem. Ges. A/B Ser..

[14] Staudinger H., Heuer W. (1934). Über hochpolymere Verbindungen, 93. Mitteil.: Über
das Zerreißen der Faden-Moleküle des Poly-styrols. Ber. Dtsch. Chem. Ges. A/B Ser..

[15] Staudinger H., Bondy H. F. (1930). Über Isopren
und Kautschuk, 19. Mitteil.: Über
die Molekülgröße des Kautschuks und der Balata. Ber. Dtsch. Chem. Ges. A/B Ser..

[16] Krusenbaum A., Grätz S., Tigineh G. T., Borchardt L., Kim J. G. (2022). The mechanochemical
synthesis of polymers. Chem. Soc. Rev..

[17] Prat D., Wells A., Hayler J., Sneddon H., McElroy C. R., Abou-Shehada S., Dunn P. J. (2016). CHEM21 selection guide of classical-
and less classical-solvents. Green Chem..

[18] Wongwilawan S., Nguyen T. S., Nguyen T. P. N., Alhaji A., Lim W., Hong Y., Park J. S., Atilhan M., Kim B. J., Eddaoudi M., Yavuz C. T. (2023). Non-solvent post-modifications with
volatile reagents for remarkably porous ketone functionalized polymers
of intrinsic microporosity. Nat. Commun..

[19] Guan J., Wang X., Du J., Liang Q., He W., Liu Y., Ma J., Zhang C., Liu J. (2023). Surface-engineered
PIM-1 membranes for facile CO2 capture. Chem.
Eng. J..

[20] Ameen A. W., Ji J., Tamaddondar M., Moshenpour S., Foster A. B., Fan X., Budd P. M., Mattia D., Gorgojo P. (2021). 2D boron nitride nanosheets
in PIM-1 membranes for CO2/CH4 separation. J.
Membr. Sci..

[21] Wang J., Zhou Y., Liu X., Liu Q., Hao M., Wang S., Chen Z., Yang H., Wang X. (2024). Design and
application of metal–organic framework membranes for gas and
liquid separations. Sep. Purif. Technol..

[22] Saini H., Kallem P., Otyepková E., Geyer F., Schneemann A., Ranc V., Banat F., Zbořil R., Otyepka M., Fischer R. A., Jayaramulu K. (2021). Two-dimensional
MOF-based liquid marbles: surface energy calculations and efficient
oil–water separation using a ZIF-9-III@PVDF membrane. J. Mater. Chem. A.

[23] Li N., Ma C., Ye M., Guo X., Qiao Z., Zhong C. (2023). Mechanochemical
synthesized amino-functionalized ultramicroporous ZIF based mixed-matrix
membranes for CO2 separation. J. Membr. Sci..

[24] Karadeniz B., Howarth A. J., Stolar T., Islamoglu T., Dejanović I., Tireli M., Wasson M. C., Moon S.-Y., Farha O. K., Friščić T., Užarević K. (2018). Benign by Design: Green and Scalable
Synthesis of Zirconium UiO-Metal–Organic Frameworks by Water-Assisted
Mechanochemistry. ACS Sustainable Chem. Eng..

[25] Głowniak S., Szczęśniak B., Choma J., Jaroniec M. (2024). Mechanochemical
Synthesis of MOF-303 and Its CO2 Adsorption at Ambient Conditions. Molecules.

[26] Lyu B., Wang M., Jiang J., Jiang Z. (2022). Molecular design of
covalent–organic framework membranes for Li+/Mg2+ separation:
Significant charge effect. J. Membr. Sci..

[27] Dawson R., Cooper A. I., Adams D. J. (2012). Nanoporous
organic polymer networks. Prog. Polym. Sci..

[28] Wan H., Yan X., Yang J., Yan G., Zhang G. (2024). How to transform microporous
organic polymers for membrane-based separation: A review. Sep. Purif. Technol..

[29] Lin S., Su M., Li X., Liang S.-x. (2024). Solvent-free mechanochemical
synthesis
of a sodium disulfonate covalent organic framework for simultaneous
highly efficient selective capture and sensitive fluorescence detection
of fluoroquinolones. Sep. Purif. Technol..

[30] Liu G., Chen H., Zhang W., Ding Q., Wang J., Zhang L. (2021). Facile mechanochemistry
synthesis of magnetic covalent organic framework
composites for efficient extraction of microcystins in lake water
samples. Anal. Chim. Acta.

[31] Wu J., Wang Y., Wu Y., Xu W., Wang J., Li S., Xu Z. (2023). Freestanding covalent
organic framework membranes with
enhanced proton perm-selectivity for flow batteries. J. Membr. Sci..

[32] Yang Y., Wang Z., Song Z., Liu D., Zhang J., Guo L., Fang W., Jin J. (2023). Thermal treated
amidoxime modified
polymer of intrinsic microporosity (AOPIM-1) membranes for high permselectivity
reverse osmosis desalination. Desalination.

[33] Liu M.-L., Chen Y., Hu C., Zhang C.-X., Fu Z.-J., Xu Z., Lee Y. M., Sun S.-P. (2024). Microporous membrane with ionized
sub-nanochannels enabling highly selective monovalent and divalent
anion separation. Nat. Commun..

[34] Feng X., Zhu J., Jin J., Wang Y., Zhang Y., Van der
Bruggen B. (2024). Polymers of intrinsic microporosity for membrane-based
precise separations. Prog. Mater. Sci..

[35] Loh C. Y., Burrows A. D., Zhang X., Xie M. (2024). Polymer of Intrinsic
Microporosity Enabled pH-Responsive Adsorptive Membrane: Selectivity
and Mechanism. ACS Appl. Eng. Mater..

[36] Loh C. Y., Huang R., Bell R., Xie M. (2024). Solvent-free mechanosynthesis
of polyethyleneimine-grafted polymers of intrinsic microporosity with
enhanced adsorption capacity. Environ. Technol.
Innovation.

[37] Budd, P. M. Polymers of Intrinsic Microporosity and Their Potential in Process Intensification. In Sustainable Nanoscale Engineering; Szekely, G. ; Livingston, A. , Eds.; Elsevier, 2020; Chapter 9, pp 231–264.

[38] Zhang P., Jiang X., Wan S., Dai S. (2015). Advancing polymers
of intrinsic microporosity by mechanochemistry. J. Mater. Chem. A.

[39] Loh C. Y., Huang R., Bell R., Xie M. (2023). Towards sustainable
synthesis: a life cycle assessment of polymer of intrinsic microporosity
(PIM-1) by green mechanosynthesis. RSC Sustainability.

[40] Budd P. M., Ghanem B. S., Makhseed S., McKeown N. B., Msayib K. J., Tattershall C. E. (2004). Polymers
of intrinsic microporosity (PIMs): robust,
solution-processable, organic nanoporous materials. Chem. Commun..

[41] Hamzehpoor E., Effaty F., Borchers T. H., Stein R. S., Wahrhaftig-Lewis A., Ottenwaelder X., Friščić T., Perepichka D. F. (2024). Mechanochemical Synthesis of Boroxine-linked Covalent
Organic Frameworks. Angew. Chem., Int. Ed..

[42] Brown N., Alsudairy Z., Behera R., Akram F., Chen K., Smith-Petty K., Motley B., Williams S., Huang W., Ingram C., Li X. (2023). Green mechanochemical synthesis of
imine-linked covalent organic frameworks for high iodine capture. Green Chem..

[43] Zhu C., Pang S., Chen Z., Bi L., Wang S., Liang C., Qin C. (2022). Synthesis of Covalent
Organic Frameworks
(COFs)-Nanocellulose Composite and Its Thermal Degradation Studied
by TGA/FTIR. Polymers.

[44] Wenger S. R., Kearns E. R., Miller K. L., D’Alessandro D. M. (2023). Green,
One-Step Mechanochemical Synthesis and Techno-economic Analysis of
UiO-66-NH2. ACS Appl. Energy Mater..

[45] Ulbricht M. (2006). Advanced functional
polymer membranes. Polymer.

[46] Guillen G. R., Pan Y., Li M., Hoek E. M. V. (2011). Preparation and Characterization
of Membranes Formed by Nonsolvent Induced Phase Separation: A Review. Ind. Eng. Chem. Res..

[47] Ren, J. ; Wang, R. Preparation of Polymeric Membranes. In Membrane and Desalination Technologies; Wang, L. K. ; Chen, J. P. ; Hung, Y.-T. ; Shammas, N. K. , Eds.; Humana Press, 2011; pp 47–100.

[48] Mulder, M. Basic Principles of Membrane Technology; Springer science & business media, 2012.

[49] Kahrs C., Schwellenbach J. (2020). Membrane formation via non-solvent induced phase separation
using sustainable solvents: A comparative study. Polymer.

[50] Russo F., Galiano F., Pedace F., Aricò F., Figoli A. (2020). Dimethyl Isosorbide As a Green Solvent for Sustainable
Ultrafiltration and Microfiltration Membrane Preparation. ACS Sustainable Chem. Eng..

[51] Russo, F. ; Vigile, M. F. ; Galiano, F. ; Figoli, A. Green Solvents for Membrane Fabrication. In Green Membrane Technologies towards Environmental Sustainability; Dumée, L. F. ; Sadrzadeh, M. ; Shirazi, M. M. A. , Eds.; Elsevier, 2023; Chapter 2, pp 9–44.

[52] Sherwood J., De bruyn M., Constantinou A., Moity L., McElroy C. R., Farmer T. J., Duncan T., Raverty W., Hunt A. J., Clark J. H. (2014). Dihydrolevoglucosenone (Cyrene) as a bio-based alternative
for dipolar aprotic solvents. Chem. Commun..

[53] Cardoso A.
P., Giacobbo A., Bernardes A. M., Ferreira C. A. (2024). Performance evaluation
of polysulfone-based membranes produced with a green solvent. J. Mater. Res..

[54] Hackett C., Hale D., Bair B., Manson-Endeboh G. s.-D., Hao X., Qian X., Wickramasinghe S. R., Thompson A. (2024). Polysulfone ultrafiltration membranes fabricated from
green solvents: Significance of coagulation bath composition. Sep. Purif. Technol..

[55] Lin S., He S., Sarwar S., Milescu R. A., McElroy C. R., Dimartino S., Shao L., Lau C. H. (2023). Spray coating polymer
substrates
from a green solvent to enhance desalination performances of thin
film composites. J. Mater. Chem. A.

[56] Arif A., Chanchaona N., Lau C. H. (2023). Comparing the environmental impacts
of using bio-renewable and fossil-derived solvent in polymer membrane
fabrications. Adv. Membr..

[57] Rasool M. A., Vankelecom I. F. J. (2019). Use
of γ-valerolactone and glycerol derivatives
as bio-based renewable solvents for membrane preparation. Green Chem..

[58] Andresen-Streichert H., Jungen H., Gehl A., Müller A., Iwersen-Bergmann S. (2013). Uptake of Gamma-Valerolactone—Detection
of Gamma-Hydroxyvaleric
Acid in Human Urine Samples. J. Anal. Toxicol..

[59] Huber G. W., Iborra S., Corma A. (2006). Synthesis
of Transportation Fuels
from Biomass: Chemistry, Catalysts, and Engineering. Chem. Rev..

[60] Jia Y.-X., Xie L., Xu X.-G., Wang M. (2025). Using γ-Valerolactone as a
Nontoxic Solvent for Fabricating Anion-Exchange Membrane via Nonsolvent-Induced
Phase Separation. J. Appl. Polym. Sci..

[61] Rasool M.
A., Vankelecom I. F. J. (2021). γ-Valerolactone
as Bio-Based Solvent for Nanofiltration
Membrane Preparation. Membranes.

[62] Lu D., Jung M., Escobar I. C., Harris T. A. L. (2025). Advances in applying
sustainable materials and manufacturing scale-up in polymeric membrane
fabrication. Curr. Opin. Chem. Eng..

[63] Hassankiadeh N. T., Cui Z., Kim J. H., Shin D. W., Lee S. Y., Sanguineti A., Arcella V., Lee Y. M., Drioli E. (2015). Microporous poly­(vinylidene
fluoride) hollow fiber membranes fabricated with PolarClean as water-soluble
green diluent and additives. J. Membr. Sci..

[64] Jung J. T., Kim J. F., Wang H. H., di Nicolo E., Drioli E., Lee Y. M. (2016). Understanding the
non-solvent induced
phase separation (NIPS) effect during the fabrication of microporous
PVDF membranes via thermally induced phase separation (TIPS). J. Membr. Sci..

[65] Marino T., Blasi E., Tornaghi S., Di Nicolò E., Figoli A. (2018). Polyethersulfone membranes prepared with Rhodiasolv
Polarclean as water soluble green solvent. J.
Membr. Sci..

[66] Randová A., Bartovská L., Morávek P., Matějka P., Novotná M., Matějková S., Drioli E., Figoli A., Lanč M., Friess K. (2016). A fundamental study
of the physicochemical properties of Rhodiasolv Polarclean: A promising
alternative to common and hazardous solvents. J. Mol. Liq..

[67] Fionah A., Oluk I., Brady L., Byrne D. M., Escobar I. C. (2024). Performance
and Environmental Assessment of Biochar-Based Membranes Synthesized
from Traditional and Eco-Friendly Solvents. Membranes.

[68] Russo F., Ursino C., Sayinli B., Koyuncu I., Galiano F., Figoli A. (2021). Advancements in Sustainable PVDF
Copolymer Membrane
Preparation Using Rhodiasolv PolarClean As an Alternative Eco-Friendly
Solvent. Clean Technol..

[69] Medina-Gonzalez Y., Aimar P., Lahitte J. F., Remigy J. C. (2011). Towards green membranes:
preparation of cellulose acetate ultrafiltration membranes using methyl
lactate as a biosolvent. Int. J. Sustainable
Eng..

[70] Rasool M. A., Van Goethem C., Vankelecom I. F. J. (2020). Green
preparation process using methyl
lactate for cellulose-acetate-based nanofiltration membranes. Sep. Purif. Technol..

[71] Zhu M., Han D., Yang S., Zhang Y., Zhang H. (2025). Fabrication
of PVDF
ultrafiltration membranes with methyl lactate: enhancing performance
through green solvent practices. Green Chem..

[72] d’Ayala G. G., Marino T., de Almeida Y. M. B., de Matos Costa A. R., da Silva L. B., Argurio P., Laurienzo P. (2024). Enhancing
Sustainability in PLA Membrane Preparation through the Use of Biobased
Solvents. Polymers.

[73] Zheng D., Hua D., Hong Y., Ibrahim A.-R., Yao A., Pan J., Zhan G. (2020). Functions
of Ionic Liquids in Preparing Membranes for Liquid Separations:
A Review. Membranes.

[74] Chen L., Kim D., de Vos W. M. (2024). Enhancing
the Separation Performance of Cellulose Membranes
Fabricated from 1-Ethyl-3-methylimidazolium Acetate by Introducing
Acetone as a Co-Solvent. Membranes.

[75] Kim D., Le N. L., Nunes S. P. (2016). The effects
of a co-solvent on fabrication
of cellulose acetate membranes from solutions in 1-ethyl-3-methylimidazolium
acetate. J. Membr. Sci..

[76] Pham T. P. T., Cho C.-W., Yun Y.-S. (2010). Environmental
fate and toxicity of
ionic liquids: A review. Water Res..

[77] Ratti R. (2014). Ionic Liquids:
Synthesis and Applications in Catalysis. Adv.
Chem..

[78] Ventura S. P. M., Gonçalves A. M. M., Sintra T., Pereira J. L., Gonçalves F., Coutinho J. A. P. (2013). Designing ionic liquids: the chemical
structure role in the toxicity. Ecotoxicology.

[79] Dong X., Lu D., Harris T. A. L., Escobar I. C. (2021). Polymers and Solvents Used in Membrane
Fabrication: A Review Focusing on Sustainable Membrane Development. Membranes.

[80] Romero A., Santos A., Tojo J., Rodríguez A. (2008). Toxicity and
biodegradability of imidazolium ionic liquids. J. Hazard. Mater..

[81] Bridge A. T., Wamble N. P., Santoso M. S., Brennecke J. F., Freeman B. D. (2024). Defect-free asymmetric Matrimid gas separation membranes
using dihydrolevoglucosenone (Cyrene) as a greener polar aprotic solvent
than traditional solvents. J. Membr. Sci..

[82] Rizqi R. A., Hartono Y. V., Shalahuddin I., Nugroho W. A., Bilad M. R., Arif C., Wibisono Y. (2023). Green synthesis
of polyvinylidene
fluoride ultrafiltration membrane with upgraded hydrophilicity. Results Mater..

[83] Yoon J., Lee J., Hong S. P., Park H.-J., Kim J., Lee J., Lee C., Oh S.-G. (2024). Fabrication of biodegradable
cellulose acetate nanofibers
containing Rose Bengal dye by electrospinning technique and their
antiviral efficacy under visible light irradiation. Chemosphere.

[84] Chai, P. ; Ku, Y. ; Chan, W. In Preparation of Enhanced Antifouling Graphene Oxide Cellulose Acetate Mixed-matrix Membrane Using Ethyl Lactate Green Solvent, AIP Conference Proceedings; AIP Publishing, 2023.

[85] Lei L., Lindbråthen A., Sandru M., Gutierrez M. T. G., Zhang X., Hillestad M., He X. (2018). Spinning Cellulose
Hollow Fibers Using 1-Ethyl-3-methylimidazolium Acetate–Dimethylsulfoxide
Co-Solvent. Polymers.

[86] Kim S., Thi H. N., Kang J., Hwang J., Kim S., Park S., Lee J.-H., Abdellah M. H., Szekely G., Lee J. S., Kim J. F. (2024). Sustainable
fabrication of solvent
resistant biodegradable cellulose membranes using green solvents. Chem. Eng. J..

[87] Otitoju T. A., Kim C.-H., Ryu M., Park J., Kim T.-K., Yoo Y., Park H., Lee J.-H., Cho Y. H. (2024). Exploring green
solvents for the sustainable fabrication of bio-based polylactic acid
membranes using nonsolvent-induced phase separation. J. Cleaner Prod..

[88] Gholami F., Zinadini S., Zinatizadeh A. A., Abbasi A. R. (2018). TMU-5 metal-organic
frameworks (MOFs) as a novel nanofiller for flux increment and fouling
mitigation in PES ultrafiltration membrane. Sep. Purif. Technol..

[89] Arthanareeswaran G., Velu S., Muruganandam L. (2011). Performance
enhancement of polysulfone
ultrafiltration membrane by blending with polyurethane hydrophilic
polymer. J. Polym. Eng..

[90] Dong X., Al-Jumaily A., Escobar I. C. (2018). Investigation of the Use of a Bio-Derived
Solvent for Non-Solvent-Induced Phase Separation (NIPS) Fabrication
of Polysulfone Membranes. Membranes.

[91] Rahimpour A., Madaeni S. S., Amirinejad M., Mansourpanah Y., Zereshki S. (2009). The effect of heat treatment of PES
and PVDF ultrafiltration
membranes on morphology and performance for milk filtration. J. Membr. Sci..

[92] Habibi S., Nematollahzadeh A. (2016). Enhanced water
flux through ultrafiltration polysulfone
membrane via addition-removal of silica nano-particles: Synthesis
and characterization. J. Appl. Polym. Sci..

[93] Cheng L., Zhou Z., Li L., Xiao P., Ma Y., Liu F., Li J. (2022). PVDF/MOFs
mixed matrix ultrafiltration membrane for
efficient water treatment. Front. Chem..

[94] Saljoughi E., Sadrzadeh M., Mohammadi T. (2009). Effect of preparation variables on
morphology and pure water permeation flux through asymmetric cellulose
acetate membranes. J. Membr. Sci..

[95] Li Z., Ren J., Fane A. G., Li D. F., Wong F.-S. (2006). Influence of solvent
on the structure and performance of cellulose acetate membranes. J. Membr. Sci..

[96] Gohari R. J., Lau W. J., Matsuura T., Ismail A. F. (2013). Fabrication and
characterization of novel PES/Fe–Mn binary oxide UF mixed matrix
membrane for adsorptive removal of As­(III) from contaminated water
solution. Sep. Purif. Technol..

[97] Zhao S., Wang Z., Wang J., Yang S., Wang S. (2011). PSf/PANI nanocomposite
membrane prepared by in situ blending of PSf and PANI/NMP. J. Membr. Sci..

[98] Van
Krevelen D. W., Hoftyzer P. J. (1967). Practical evaluation of the [η]–M
relationship. J. Appl. Polym. Sci..

[99] Scott G. (1992). Properties
of polymers. Their correlation with chemical structure; their numerical
estimation and prediction from additive group contributions. Endeavour.

[100] Nardella F., Prothmann J., Sandahl M., Spégel P., Ribechini E., Turner C. (2023). Native lignin extraction from soft-
and hardwood by green and benign sub/supercritical fluid extraction
methodologies. RSC Adv..

[101] Morales-Jiménez M., Palacio D. A., Palencia M., Meléndrez M. F., Rivas B. L. (2023). Bio-Based Polymeric
Membranes: Development
and Environmental Applications. Membranes.

[102] Cheng H. N., Dowd M. K., Selling G. W., Biswas A. (2010). Synthesis
of cellulose acetate from cotton byproducts. Carbohydr. Polym..

[103] El-Sheikh M. A., El-Rafie S. M., Abdel-Halim E. S., El-Rafie M. H. (2013). Green Synthesis of Hydroxyethyl Cellulose-Stabilized
Silver Nanoparticles. J. Polym..

[104] Li S., Wang D., Xiao H., Zhang H., Cao S., Chen L., Ni Y., Huang L. (2021). Ultra-low pressure
cellulose-based nanofiltration membrane fabricated on layer-by-layer
assembly for efficient sodium chloride removal. Carbohydr. Polym..

[105] P A., K H., S M., Pius A. (2024). Cellulose acetate based-membrane
supported by metal-organic frameworks for the removal of diclofenac
and ciprofloxacin from polluted water. Groundwater
Sustainable Dev..

[106] Ning D., Lu Z., Tian C., Yan N., Xie F., Li N., Hua L. (2023). Superwettable cellulose acetate-based
nanofiber membrane with spider-web structure for highly efficient
oily water purification. Int. J. Biol. Macromol..

[107] Torkashvand J., Saeedi-Jurkuyeh A., Kalantary R. R., Gholami M., Esrafili A., Yousefi M., Farzadkia M. (2022). Preparation
of a cellulose acetate membrane using cigarette butt recycling and
investigation of its efficiency in removing heavy metals from aqueous
solution. Sci. Rep..

[108] Song Z., Chen R., Luo S., Yu W., Yuan J., Lin F., Wang M., Cao X., Liao Y., Huang B., You X. (2024). Regenerated cellulose
membranes for efficient separation of organic mixtures. Sep. Purif. Technol..

[109] Salehi E., Khajavian M., Sahebjamee N., Mahmoudi M., Drioli E., Matsuura T. (2022). Advances in nanocomposite
and nanostructured chitosan membrane adsorbents for environmental
remediation: A review. Desalination.

[110] Vedula S. S., Yadav G. D. (2021). Chitosan-based membranes
preparation
and applications: Challenges and opportunities. J. Indian Chem. Soc..

[111] Wu J., Dong Z., Li X., Li P., Wei J., Hu M., Geng L., Peng X. (2022). Constructing
acid-resistant chitosan/cellulose
nanofibrils composite membrane for the adsorption of methylene blue. J. Environ. Chem. Eng..

[112] Wang Y., Li H., Zhao J., Li Z., Li J., Li J. (2024). Polyethyleneimine functionalized
chitosan nanofiltration
membrane for nuclear wastewater decontamination. J. Ind. Eng. Chem..

[113] Xia Y., Wang Z., Chen L.-Y., Xiong S.-W., Zhang P., Fu P.-G., Gai J.-G. (2020). Nanoscale polyelectrolyte/metal
ion
hydrogel modified RO membrane with dual anti-fouling mechanism and
superhigh transport property. Desalination.

[114] Wang Y., He Y., Yu J., Zhang L., Li S., Li H. (2022). Alginate-based nanofibrous
membrane with robust photo-Fenton
self-cleaning property for efficient crude oil/water emulsion separation. Sep. Purif. Technol..

[115] Thu N. T. T., Hoang L. H., Cuong P. K., Viet-Linh N., Nga T. T. H., Kim D. D., Leong Y. K., Nhi-Cong L. T. (2023). Evaluation
of polyhydroxyalkanoate (PHA) synthesis by Pichia sp. TSLS24 yeast
isolated in Vietnam. Sci. Rep..

[116] Dana H. R., Ebrahimi F. (2023). Synthesis, properties,
and applications
of polylactic acid-based polymers. Polym. Eng.
Sci..

[117] Vatanpour V., Dehqan A., Paziresh S., Zinadini S., Zinatizadeh A. A., Koyuncu I. (2022). Polylactic acid in the fabrication
of separation membranes: A review. Sep. Purif.
Technol..

[118] Kaseem M., Ur Rehman Z., Hossain S., Singh A. K., Dikici B. (2021). A Review on
Synthesis, Properties, and Applications
of Polylactic Acid/Silica Composites. Polymers.

[119] Zaaba N. F., Jaafar M. (2020). A review on degradation mechanisms
of polylactic acid: Hydrolytic, photodegradative, microbial, and enzymatic
degradation. Polym. Eng. Sci..

[120] Zhang J., Jiao Y., Zhang Y., Wang K., Sui X., Song D., Drioli E., Cheng X. (2022). Development of Hydrophilic
Polylactic Acid Hollow-Fiber Membranes for Water Remediation. Ind. Eng. Chem. Res..

[121] Khalil H., Hegab H. M., Nassar L., Wadi V. S., Naddeo V., Yousef A. F., Banat F., Hasan S. W. (2022). Asymmetrical
ultrafiltration membranes based on polylactic acid for the removal
of organic substances from wastewater. J. Water
Process Eng..

[122] Nassar L., Wadi V. S., Hegab H. M., Khalil H., Banat F., Naddeo V., Hasan S. W. (2022). Sustainable and
green polylactic acid-based membrane embedded with self-assembled
positively charged f-MWCNTs/GO nanohybrids for the removal of nutrients
from wastewater. npj Clean Water.

[123] Tomietto, P. ; Loulergue, P. ; Paugam, L. ; Audic, J.-L. Polyhydroxyalkanoates (PHAs) for the Fabrication of Filtration Membranes. In Advances in Science, Technology Innovation; Zhang, Z. ; Zhang, W. ; Chehimi, M. M. , Eds.; Springer International Publishing, 2021; pp 177–195.

[124] Mekonnen T., Mussone P., Khalil H., Bressler D. (2013). Progress in
bio-based plastics and plasticizing modifications. J. Mater. Chem. A.

[125] Anjum A., Zuber M., Zia K. M., Noreen A., Anjum M. N., Tabasum S. (2016). Microbial production
of polyhydroxyalkanoates
(PHAs) and its copolymers: A review of recent advancements. Int. J. Biol. Macromol..

[126] Masood F., Yasin T., Hameed A. (2015). Polyhydroxyalkanoates
– what are the uses? Current challenges and perspectives. Crit. Rev. Biotechnol..

[127] Tomietto P., Carré M., Loulergue P., Paugam L., Audic J.-L. (2020). Polyhydroxyalkanoate
(PHA) based
microfiltration membranes: Tailoring the structure by the non-solvent
induced phase separation (NIPS) process. Polymer.

[128] Sariipek F. B., Gündoğdu Y., Kiliç H. Ş. (2023). Fabrication of eco-friendly superhydrophobic
and superoleophilic
PHB-SiO bionanofiber membrane for gravity-driven oil/water separation. J. Appl. Polym. Sci..

[129] Bang J., Park S., Hwang S.-W., Oh J.-K., Yeo H., Jin H.-J., Kwak H. W. (2023). Biodegradable
and hydrophobic nanofibrous
membranes produced by solution blow spinning for efficient oil/water
separation. Chemosphere.

[130] Ling S., Qin Z., Huang W., Cao S., Kaplan D. L., Buehler M. J. (2017). Design and function of biomimetic
multilayer water purification membranes. Sci.
Adv..

[131] Xing X., Han Y., Cheng H. (2023). Biomedical applications
of chitosan/silk fibroin composites: A review. Int. J. Biol. Macromol..

[132] Zhang Y., Peng S., Li X., Wang X., Jiang J., Liu X., Wang L. (2023). Design and
function
of lignin/silk fibroin-based multilayer water purification membranes
for dye adsorption. Int. J. Biol. Macromol..

[133] Lee S., Kim H.-J., Tian M., Khang G., Kim H.-W., Bae T.-H., Lee J. (2023). Silk fibroin-coated
polyamide thin-film
composite membranes with anti-scaling properties. Desalination.

[134] Chowdhury, S. R. ; Mh Busra, M. F. ; Lokanathan, Y. ; Ng, M. H. ; Law, J. X. ; Cletus, U. C. ; Binti Haji Idrus, R. Collagen Type I: A Versatile Biomaterial. In Advances in Experimental Medicine and Biology; Chun, H. J. ; Park, K. ; Kim, C.-H. ; Khang, G. , Eds.; Springer Singapore, 2018; pp 389–414. 10.1007/978-981-13-0947-2_21 30357700

[135] Desiriani R., Susanto H., Aryanti N., Abriyanto H. (2023). Improvement
of the antifouling and antibacterial properties of polyethersulfone
membrane by incorporating the natural additives collagen and green
tea. Results Eng..

[136] Mohammadi R., Mohammadifar M. A., Mortazavian A. M., Rouhi M., Ghasemi J. B., Delshadian Z. (2016). Extraction
optimization of pepsin-soluble collagen from eggshell membrane by
response surface methodology (RSM). Food Chem..

[137] Alias, N. H. ; Abdullah, N. ; Othman, N. H. ; Marpani, F. ; Zainol, M. M. ; Shayuti, M. S. M. Sustainability Challenges and Future Perspectives of Biopolymer. In Biopolymers: Recent Updates, Challenges and Opportunities; Nadda, A. K. ; Sharma, S. ; Bhat, R. , Eds.; Springer International Publishing, 2022; pp 373–389.

[138] Barletta M., Aversa C., Ayyoob M., Gisario A., Hamad K., Mehrpouya M., Vahabi H. (2022). Poly­(butylene succinate)
(PBS): Materials, processing, and industrial applications. Prog. Polym. Sci..

[139] Kartik A., Akhil D., Lakshmi D., Gopinath K. P., Arun J., Sivaramakrishnan R., Pugazhendhi A. (2021). A critical
review on production of biopolymers from algae biomass and their applications. Bioresour. Technol..

[140] Torre-Celeizabal A., Russo F., Galiano F., Figoli A., Casado-Coterillo C., Garea A. (2025). Green Synthesis of
Cellulose Acetate
Mixed Matrix Membranes: Structure–Function Characterization. ACS Sustainable Chem. Eng..

[141] Commission, E. . REACH Regulation. 2023.

[142] Agency, E. P. . Toxic Substances Control Act of 1976. 2016.

[143] Agency, E. P. . Clean Water Act. 2018.

[144] Organisation, I. S. . ISO 14001:2015. 2015.

[145] Commission, E. . Circular Economy Action Plan. 2020.

[146] Landaburu-Aguirre J., García-Pacheco R., Molina S., Rodríguez-Sáez L., Rabadán J., García-Calvo E. (2016). Fouling prevention, preparing for
re-use and membrane
recycling. Towards circular economy in RO desalination. Desalination.

[147] Grossi L. B., da Silva B. R. S., Neves E. F. O., Lange L. C., Amaral M. C. S. (2021). Reverse osmosis elements waste assessment: Screening
and forecasting of emerging waste in Brazil. Desalination.

[148] García-Pacheco R., Landaburu-Aguirre J., Molina S., Rodríguez-Sáez L., Teli S. B., García-Calvo E. (2015). Transformation of end-of-life RO
membranes into NF and UF membranes: Evaluation of membrane performance. J. Membr. Sci..

[149] Liu C., Wang W., Yang B., Xiao K., Zhao H. (2021). Separation,
anti-fouling, and chlorine resistance of the polyamide reverse osmosis
membrane: From mechanisms to mitigation strategies. Water Res..

[150] de Paula E. C., Gomes J. C. L., Amaral M. C. S. (2017). Recycling of
end-of-life reverse osmosis membranes by oxidative treatment: a technical
evaluation. Water Sci. Technol..

[151] Fujioka T., Ngo M. T. T., Boivin S., Kawahara K., Takada A., Nakamura Y., Yoshikawa H. (2020). Controlling
biofouling and disinfection by-product formation during reverse osmosis
treatment for seawater desalination. Desalination.

[152] Wang H., Xu Y., Ma B., Zou W., Zeng J., Dai R., Wang Z. (2024). Alkaline pre-treatment
enables controllable downcycling of Si-Al fouled end-of-life RO membrane
to NF and UF membranes. J. Membr. Sci..

[153] Moreira V. R., Grossi L. B., Guimaraes R. N., Amaral M. C. S. (2024). Recycled nanofiltration membrane as a low-cost alternative
to remove uranium from drinking water in remote communities. Desalination.

[154] Cui J., Chen Y., Guo P., Su W., Xu L., Zhang Y. (2023). Recycling End-of-Life RO Membranes for NF Membranes
via Layer-by-Layer
Assembly and Interfacial Polymerization. Ind.
Eng. Chem. Res..

[155] Dai R., Han H., Wang T., Li J., Wu Z., Tang C. Y., Wang Z. (2021). Cleaning–Healing–Interfacial
Polymerization Strategy for Upcycling Real End-of-Life Polyvinylidene
Fluoride Microfiltration Membranes. ACS Sustainable
Chem. Eng..

[156] Wang X., Han H., Zhou H., Wang T., Dai R., Wang Z. (2022). Rapid Upcycling of End-of-Life Microfiltration Membrane
Mediated by the Healing of Metal–Organic Complex. ACS Sustainable Chem. Eng..

[157] Dai R., Han H., Wang T., Li J., Tang C. Y., Wang Z. (2021). Fouling is the beginning: upcycling
biopolymer-fouled substrates
for fabricating high-permeance thin-film composite polyamide membranes. Green Chem..

[158] Dai R., Chen J., Han H., Zhou H., Wang Z. (2023). Interfacial
Wettability Regulation Enables One-Step Upcycling of the End-of-Life
Polymeric Microfiltration Membrane. ACS ES&T
Eng..

[159] Li B., Wang S., Loh X. J., Li Z., Chung T.-S. (2023). Closed-loop
recyclable membranes enabled by covalent adaptable networks for water
purification. Proc. Natl. Acad. Sci. U.S.A..

[160] Li B., Qu C., Wang S., Yeo J. C. C., Surat’man N. E. B., Loh X. J., Li Z., Chung T.-S. (2024). Closed-loop recyclable
dynamic covalent crosslinked nanofibrous membranes for efficient oil/water
separation. J. Membr. Sci..

[161] Poniatowska J., Houben M., Borneman Z., Nijmeijer K. (2025). Towards organic
solvent nanofiltration (OSN) membrane recyclability through Diels-Alder
(DA) dynamic covalent bonds. Sep. Purif. Technol..

[162] Ramírez-Martínez M., Aristizabal S., Upadhyaya L., Emwas A.-H., Hadjichristidis N., Nunes S. P. (2024). Recyclable Membranes through Reversible and Dynamic
Crosslinking. ACS Appl. Polym. Mater..

[163] Gupta R. S., Mandal S., Malakar A., Rege S., Islam S. S., Samanta K., Misra A., Bose S. (2023). Graphene oxide
offers precise molecular sieving, structural integrity, microplastic
removal, and closed-loop circularity in water-remediating membranes
through a covalent adaptable network. J. Mater.
Chem. A.

[164] Wang S., Wang N., Kai D., Li B., Wu J., Yeo J. C. C., Xu X., Zhu J., Loh X. J., Hadjichristidis N., Li Z. (2023). In-situ forming dynamic covalently
crosslinked nanofibers with one-pot closed-loop recyclability. Nat. Commun..

[165] de Paula E. C., Amaral M. C. S. (2018). Environmental and economic evaluation
of end-of-life reverse osmosis membranes recycling by means of chemical
conversion. J. Cleaner Prod..

[166] Chen J., Dai R., Wang Z. (2023). Closing the
loop of
membranes by recycling end-of-life membranes: Comparative life cycle
assessment and economic analysis. Resour., Conserv.
Recycl..

[167] Reiser P., Neubert M., Eberhard A., Torresi L., Zhou C., Shao C., Metni H., van Hoesel C., Schopmans H., Sommer T., Friederich P. (2022). Graph neural
networks for materials science and chemistry. Commun. Mater..

